# Circuits and Biomarkers of the Central Nervous System Relating to Astronaut Performance: Summary Report for a NASA-Sponsored Technical Interchange Meeting

**DOI:** 10.3390/life13091852

**Published:** 2023-08-31

**Authors:** Joshua S. Alwood, Ajitkumar P. Mulavara, Janani Iyer, Siddhita D. Mhatre, Susanna Rosi, Mark Shelhamer, Catherine Davis, Christopher W. Jones, Xiao Wen Mao, Rajeev I. Desai, Alexandra M. Whitmire, Thomas J. Williams

**Affiliations:** 1 NASA Ames Research Center, Moffett Field, CA 94035, USA; 2 KBR, Houston, TX 77058, USA; 3 Universities Space Research Association (USRA), Moffett Field, CA 94035, USA; 4 Department of Physical Therapy & Rehabilitation Science, University of California, San Francisco, CA 94110, USA; 5 Department of Neurological Surgery, University of California, San Francisco, CA 94110, USA; 6 Department of Otolaryngology–Head and Neck Surgery, Johns Hopkins University, Baltimore, MD 21205, USA; 7 Department of Pharmacology and Molecular Therapeutics, Uniformed Services University of the Health Sciences (USUHS), Bethesda, MD 20814, USA; 8 Department of Psychiatry, Perelman School of Medicine, University of Pennsylvania, Philadelphia, PA 19104, USA; 9 Department of Basic Sciences, Division of Biomedical Engineering Sciences (BMES), Loma Linda University Health, Loma Linda, CA 92354, USA; 10 Integrative Neurochemistry Laboratory, Behavioral Biology Program, McLean Hospital-Harvard Medical School, Belmont, MA 02478, USA; 11 NASA Johnson Space Center, Houston, TX 77058, USA

**Keywords:** biomarker, cognition, behavior, performance, brain circuit, astronaut, CNS

## Abstract

Biomarkers, ranging from molecules to behavior, can be used to identify thresholds beyond which performance of mission tasks may be compromised and could potentially trigger the activation of countermeasures. Identification of homologous brain regions and/or neural circuits related to operational performance may allow for translational studies between species. Three discussion groups were directed to use operationally relevant performance tasks as a driver when identifying biomarkers and brain regions or circuits for selected constructs. Here we summarize small-group discussions in tables of circuits and biomarkers categorized by (a) sensorimotor, (b) behavioral medicine and (c) integrated approaches (e.g., physiological responses). In total, hundreds of biomarkers have been identified and are summarized herein by the respective group leads. We hope the meeting proceedings become a rich resource for NASA’s Human Research Program (HRP) and the community of researchers.

## 1. Introduction

Astronauts on long-duration space missions (e.g., transits to Mars) will experience the combined, potentially synergistic, impacts of simultaneous exposures to spaceflight hazards that affect the central nervous system (CNS) and operationally relevant behavior and performance [1]. While individual spaceflight hazards are often individually well quantified, in long-duration spaceflight, astronauts will experience multiple hazards simultaneously [2,3].

Parcelsus’ famous dictum on dose effects of exposures [4] reinforces the importance of an integrated approach to systematically identify and investigate the relationships of how spaceflight exposures may synergistically interact to pose a risk to the astronauts and the mission. NASA developed the Combined Behavioral Stressors (CBS) project which integrates research topics across three high-impact spaceflight hazard exposures—space radiation, isolation & confinement, and altered gravity—to inform performance outcome limits and permissible exposure limits, and to help identify and establish mitigation strategies. An integrated research approach is focused on identifying biomarker changes associated with exposures to the CBS-associated hazards to identify and develop effective monitoring, and apply countermeasures for mitigating risk to crew health and performance [5]. This is consistent with recent calls for more comprehensive and integrated biomarkers to better identify how different biomarkers can exert different causal effects between and among them [6].

The CBS Integrated Research Plan identifies biomarkers that are linked to in-flight and post-flight decrements in an astronaut’s operational performance resulting from simultaneous exposures to the CBS-relevant spaceflight hazards. In this context, a biomarker is defined as a characteristic that is “objectively measured and evaluated as an indicator of normal biologic processes, pathogenic processes, or pharmacologic responses to a therapeutic intervention” [7].

As sampling of in situ biomarkers in astronauts is not necessarily possible, translational models are useful. To promote the utility of translational models, NASA consistently updates the exposure levels in rodents as they relate to humans; for example, NASA recently adjusted their integrated research platforms involving animal exposures to expected levels of spaceflight radiation related to dose and duration [8]. It is, therefore, essential that biomarkers are useful for bi-directional translation of homologous human and animal measures, which is a cornerstone of the NASA’s CBS project—allowing for the linking of the probability for performance decrements (during and/or after mission) to the level of exposure to a CBS relevant spaceflight hazard, such as radiation exposure.

This paper reviews the results of NASA’s biomarker technical interchange meeting (TIM) that was focused on creating a comprehensive list of constructs, identifying underlying and related brain regions, neural circuits, and biomarkers for inclusion in predictive models to assess and validate changes in future astronaut risk status, as well as to identify changes in operationally relevant brain pathways (e.g., procedural memory) after exposures to varying types and amounts of potentially synergistically acting spaceflight hazards. The overall goals of this biomarker TIM were to (i) identify relevant brain regions, neural circuits, functions, and associated biomarkers, and relate them to operationally relevant performance, and (ii) identify any critical needs for new biomarker knowledge (“gaps”) that can be filled by additional focused and translational animal experiments that include a plausible pathway toward eventual biomarker validation in humans.

## 2. Meeting Synopsis

Biomarkers—ranging from molecules to behavior—can be used to identify thresholds beyond which performance of mission tasks may be compromised and could potentially trigger the activation of countermeasures. Identification of homologous brain regions and/or neural circuits related to operational performance may allow for translational studies between species. Three discussion groups were asked to use operationally relevant performance tasks as drivers when identifying biomarkers and regions or circuits for the constructs listed in Appendix A. Participants are listed in Appendix B. Here, we summarize the discussions below across the three groups. In total, hundreds of biomarkers have been identified, with references provided mainly in the respective tables for each group. We hope the meeting proceedings become a rich resource for NASA’s Human Research Program (HRP) and the community of researchers.

## 3. Summaries of Discussions and Recommendations from Each of the Breakout Sessions

### 3.1. Sensorimotor Influences on Operational Performance (Leads: S. Rosi, M. Shelhamer)

The goal of Group 1 was to create lists of biomarkers and brain regions and/or neural circuits related to operational performance for constructs that are prioritized in HRP’s sensorimotor risk. Group 1 assessed the following 13 key constructs in Table 1: visual function, spatial orientation, vestibular, proprioception, hearing, motion sickness, smell and taste, postural control and balance, locomotion, fine motor control, perception, gaze, and pain. Note that the panel assessed translatability based on the existence of rodent models and did not suggest using non-human primates (NHPs), nor did they identify a construct that should be tested in NHPs.

#### 3.1.1. Summary of Discussions

During discussion of each of the 13 constructs, 10 themes emerged. Although identification of themes was outside the scope of the panel, these themes were applicable to nearly all constructs discussed and, therefore, we define them here:

1. Connections between constructs. Distinctions between the constructs are, in many cases, artificial. Although segregated disciplinary expertise has achieved a great deal in the sensorimotor domain, the different constructs are so closely interconnected that it is hard to discuss them separately in a way that is true to the science and to the operational implications. As an example, vestibular function, gaze control, balance, and locomotion are very closely related, and yet they are often addressed as specific and separable. Another example is perception. Almost all sensorimotor constructs involve perception in some way; vestibular perception—perception of the upright—affects the ability to balance. Perception of upright is influenced by changes that occur in microgravity, which is a vestibular effect. Again, these specific constructs become tightly entangled and it is difficult to separate them in terms of biomarkers and operational relevance.

2. Many spaceflight stressors and sensorimotor effects occur simultaneously with different time courses. Not only do the different constructs interact, they do so with different time courses. The most overt and acute forms of vestibular adaptation (related to space motion sickness) occur over the course of a few days, whereas other vestibular-mediated functions (e.g., the sense of being truly comfortable with the three-dimensional aspects of motion in a weightless environment) develop over several weeks. Some adaptive sensorimotor changes in space occur with similar time courses as those seen in analogous environments on the ground. For example, the changing contributions of vestibular, proprioceptive, and efference copy information during recovery from labyrinthectomy in an animal model [9] have time courses that mimic recovery of motor control during locomotion after spaceflight [10]. Similarly, ground-based studies in animals show that development of efference copy over several weeks mimics the time course of the development of three-dimensional spatial sense in astronauts over the same time period. The similar time courses suggest that these may be aspects of the same underlying process. This might provide translational opportunities from ground-based animal models and may inform a process for preadaptation paradigms for spaceflight.

3. Multi-sensory integration. This is related to the theme of interacting constructs. Most sensorimotor behaviors and perceptions arise from the simultaneous activation of multiple sensory systems. An obvious example is the combination of visual and vestibular information for gaze control (vestibulo-ocular reflex (VOR)). Another is the prevalence of proprioceptive and kinesthetic influences, in addition to vestibular and visual influences, on posture and locomotion.

4. Stress. Spaceflight involves multiple simultaneous stressors—physiological, psychological, and environmental. These have widespread and sometimes unknown influences on sensorimotor function, and likely on the ability to adaptively alter sensorimotor function. The effects of stress on motor learning and on motion sickness are two examples: stress affects motor learning, which alters adaptation, which can change the ability to recover from motion sickness, which can increase stress.

5. Learning. Almost all the individual constructs exhibit adaptive behaviors to spaceflight and these adaptive behaviors may complicate the usefulness of the constructs as biomarkers because the response that is being assessed will change with adaptation to spaceflight. Of course, such adaptation is desirable and should be promoted, but it complicates the use of a biomarker to identify increased risk to astronaut health and performance. This would be especially true in missions of extended duration where the adaptive processes might not be understood. A specific biomarker for learning and adaptation would be desirable.

6. Some constructs might be easily measured but lack relevance. As an example, the angular VOR has been extensively studied and is easy to measure, but little or no evidence exists that it changes significantly due to spaceflight, or that any changes have an operational impact.

7. Neural circuits. Interpretation of neural circuitry is not always straightforward. There is not always a direct analogy between animals (where many circuits have been delineated) and humans; the neural circuitry is different in some cases, and there are also adaptive changes that make the definition of standard circuits difficult. Circuit function is implicitly assessed with behavioral measures, so knowledge of some circuit characteristics such as neurotransmitters and common pathways might aid in the interpretation of behavioral markers.

8. Vestibular Cognition. The relationship between cognition and the vestibular system, and the vestibular effects on cognition, is operationally relevant and directly connects cognition and sensorimotor functions. This connection is seen in many patients with vestibular problems. No specific construct exists for this, and it is difficult to conceive of a specific biomarker.

Overall, the sensorimotor issues of multi-sensory/multi-effector interactions and learning, and their relation to stress, are not yet sufficiently studied, and they likely greatly influence human performance in space. These do not yet lend themselves to direct biomarker identification.

#### 3.1.2. Recommendations

The panel evaluated each specific construct to determine if a good biomarker exists that is operationally relevant for astronauts, and that translates from animal models. The panel also commented on gaps in each construct that would need to be filled to produce an effective biomarker.

1. Visual function is easily measured (acuity, visual fields, etc.), and these measures may help to parse out visual effects from motor effects when there is a functional deficit. Retinal remodeling can be assessed with optical coherence tomography (in flight), and is hence a biomarker. Translatability is clear because many of these aspects can be tested in rodents (e.g., visual acuity in mice and even real-time visual tracking). This is clearly a useful biomarker.

2. Spatial orientation is extremely important. The panel extensively discussed grid cells—the cells in the entorhinal cortex that underlie spatial orientation. The firing of grid cells provides information that can be used to assess spatial orientation as it adapts to alterations in gravity, which is further substantiated as a potential biomarker due to its translational potential as grid cells are present and accessible in rodents. Thus, neural circuits in the hippocampus and medial entorhinal cortex are important.

3. A great deal of information exists on vestibular function in spaceflight. Basic vestibular function is not significantly altered in the microgravity environment of space, although central processing and higher-level derived functions (e.g., spatial orientation, tilt-translation perception) often are. It is, however, important to consider vestibular changes in the context of the integrated spaceflight stressors. So, as noted, the VOR changes little in weightlessness, but it would be useful to assess VOR in the context of other stressors (e.g., radiation, fatigue, etc.); for example, what is the combined impact of multiple stressors? These aspects need to be elucidated, which can be accomplished through rodent studies (e.g., the narrow balance beam as a viable animal assessment). Taken together, vestibular change (e.g., VOR or balance beam performance) is a suitable biomarker.

4. Proprioception was identified as one of the most strongly interconnected constructs, exhibiting significant overlap with several other constructs. Little is known about the effects of (CBS risks) radiation or other stressors on the peripheral nervous system and, consequently, proprioception (this is a gap in knowledge). A rodent model would provide translational opportunities, as proprioception can be measured in that model (e.g., tape removal test, whisker test). Hence, measures of proprioception are suitable biomarkers.

5. Hearing loss is often a factor associated with spaceflight, perhaps due in part to fluid shifts, and hearing assessment in flight may help to parse out the effect of the fluid shift from noise-induced loss. However, the panel noted that these data are not particularly operationally relevant: hearing loss has not been a functional problem. As such, hearing loss is not a priority biomarker.

6. Motion sickness is a known problem that needs to be further assessed because it can have serious operational impacts [11,12], especially when first experiencing a gravity field after extended weightlessness. Motion sickness susceptibility is still unpredictable. This line of work might be revisited with more recent knowledge on learning and adaptation or might be investigated in relation to the impact on specific operational tasks. We do not know how motion sickness induces stress and how stress feeds back to motion sickness and the overall well-being of astronauts. The interaction of motion sickness, sopite, stress, and crew performance has been studied in other contexts. This work should be reviewed; however, it may still be valuable to investigate these effects in the specific context of spaceflight, with its multiple simultaneous stressors and unique demands. Again, there are several overlapping biomarkers. A drawback in this area is translatability, because it is very difficult to measure motion sickness in rodents. This is a useful biomarker, albeit with some uncertainties as to translational aspects.

7. Smell and taste are particularly important for humans as social creatures and are also clearly important in space. These constructs overlap with the well-being and operational performance of astronauts. Smell and olfaction can be markers for neurodegeneration. Loss of olfaction (anosmia) is an early marker in COVID-19 and Alzheimer’s disease, as examples, and is therefore a biomarker for neurodegeneration that can also easily be tested in rodents. This biomarker is rated highly.

8. Posture and balance are important operational issues. They are problematic as biomarkers because, again, their functions cannot be isolated to discrete neural circuits due to the overlap of several circuits for multi-sensory integration and motor control. Rodent models are somewhat problematic because of the difference between neural circuits and functions in organisms with four legs (rodents) relative to two legs (humans).

9. As with posture and balance, locomotion is operationally relevant and important, but good rodent models in spaceflight or microgravity environments are lacking. It might be useful to consider static/dynamic balance control as opposed to posture/locomotion.

10. Fine motor control is difficult to assess because of the large number of confounders. Related factors that can alter fine motor function include changes in proprioception, hand-eye coordination, and others. Although functionally important, it may not be particularly relevant for operational control tasks, and suitable rodent models are lacking. The many confounders alone make this problematic as a discrete biomarker.

11. Perception is in fact a component of almost all the other constructs because it can include spatial orientation, depth perception, vestibular orientation, time perception, and others. Understanding of this construct is important and would address many of the other constructs, but there are many overlaps. Proprioception may be altered and is a critical issue on its own, but it will be most important to address in the context of other stressors. Specific aspects of perception have been noted in spaceflight and can have operational impacts, and so it would also be beneficial to consider perception in this performance context. Nevertheless, parsing out perceptual effects per se remains difficult. Thus, this was not considered to be a good biomarker.

12. The panel did not rate gaze and pain highly as biomarkers. Gaze largely overlaps vestibular function (and has been studied almost as much), so gaze control can be subsumed under vestibular function. Pain per se is not a good biomarker because of confounders between the perception and the sensation of pain. Nociception can depend on sex and other individual factors. Although biomarkers of inflammation exist, these are associated with pain. Hence, pain itself is not a discrete biomarker.

**Table 1:** Circuits and biomarkers for sensorimotor domains.

Key Indicator/Construct	Human Performance Test (Details about the Actual Test/Assay)	Animal Performance Test (Details about the Actual Test/Assay)	Caveats/Notes/Related Functional Performance Tasks/Prediction of Behavioral Outcome in Humans	Brain Region	Human/NHP Neural Circuit/Pathways	Rodent Neural Circuit/Pathway	Biomarkers (Rodents/Humans/NHPs)		Gaps/Notes
							Inaccessible	Accessible (Translatable to Astronauts)	
**Visual**	Visual field testing	Visual field testing		Visual cortex (Occipital lobe of the primary cortex)	Retino-geniculate-striate pathway (Conscious vision) Dorsal pathway (spatial location and action): Retina → LGN →V1 → V2 → MT (parietal lobe) Ventral pathway (characteristics of objects): Retina →LGN →V1 → V2 → V4 (temporal lobe) [13]	Retina-Superior Colliculus-Lateral posterior nucleus-Visual cortex1 pathway [14]	Retinal markers-autopsy, superior colliculus pathway—neural circuitry, intracranial pressure in astronauts—lumbar puncture for pressure detection, retinal vasculature imaging—vessel length density and loss of photo receptor cells, role of endothelial structure or vasculature, acceleration of incident of cataract (on cornea, not CNS) and light flashes (post-flight and long-term issue), fluorescent imaging of the retinal vasculature.	Imaging: Inflight CT, MRI imaging, ultrasound, OCT, visual field measurements, cataract as predictor Structural changes in eye, nerve, occipital cortex, pretectum, superior colliculus. Vision function test, sampling of tears [15], Intraocular pressure measurement, Saccades [16], Behavioral measures, Live pupil tracking	(1) Potential Optical/Eye damage in astronauts—could also be indicator of neurological symptoms. (2) Any imaging other than ultrasound is difficult to do in space. Difficult to get a gold standard test for intracranial pressure in space. (3) Possibility of lumbar punctures in astronaut—intracranial pressure. (4) VR environments for complex sensory integration—Somatosensory component
**Spatial Orientation**	1. Path integration-passive and active 2. Virtual maze perspective taking tests 3. Visual object learning (VOLT)	1. Changes in activity of head direction, grid, place cells 2. Morris water maze 3. Spatial navigation 4. Touch screen cognitive testing [17].	- Test in higher animals: NHP -Spatial navigation	Hippocampus and parahippocampal regions, cerebellum, brain stem, Retrosplenial cortex (Grid cells, border cells, head direction cells—cortical regions- egocentric and allocentric reference frame) [18]	Vestibulospinal pathway	Proposed head direction pathway 1: Vestibular nuclei (VN) → Cerebellum → ventral lateral nucleus of thalamus (VLN) → parietal cortex → temporal cortex → hippocampus? Proposed head direction pathway 2: Vestibular nuclei (VN) → hippocampus [19]	Hippocampal protein lysate: Afg3l1, Tpx2, Neuroligin-3, RB1-inducible coiled-coil 1, Mast3, Kif21a, DnaJ (Hsp40) homolog, SLIT-ROBO Rho GTPase-activating protein 2, Rasgrf1 [20]	Structural changes in hippocampus, anterior thalamus, subiculum. Electrodermal activity measured by wrist worn device [21], Optical coherence tomography (OCT), Illusionary experience, somatographic illusion—questionnaire	(1) Virtual reality biomarker development for astronauts. (2) Spatial orientation during g-transitions (3) Different species have varied responses. Need a model that would be most translatable.
**Vestibular**	1. Drop test/Jump down test 2. VEMP 3. OVAR response (Sensorimotor component after 30 rpm) 4. Time constant or constant rotation 5. ocular counter roll (but noisy)	1. Balance beam test (narrow beam) Righting reflex 2. VEMP (can be done in space and can help distinguish utricular and saccular functions) 3. OVAR response 4. Active vs. Passive motion on vestibular nucleus neurons 5. VSEP (otolith function) 6. Swimming test (for subtle deficits, screening test)	Test in higher animals: NHP	Thalamus and cortex	Thalamocortical pathways Anterior vestibulothalamic pathway: Vestibular nuclei (VN) → Nucleus prepositus and supragenual nucleus (NPH/SGN) → Anterior dorsal thalamus (ADN) → Entorhinal cortex → Hippocampus Posterior vestibulothalamic pathway: Vestibular nuclei (VN) → Ventral posterior lateral nucleus (VPL) → vestibular cortical areas. [9] -Three neuron pathway Vestibulo-ocular reflex: vestibular afferents → vestibular nuclei → Vestibulo-ocular reflex and efferent (vestibular processing)	(1) Vestibular nucleus → Dorsal tegmental nucleus (DTN) → Lateral mammillary nucleus (LMN) → Anterodorsal nucleus (ADN) → Post-subiculum (PS) → Hippocampus (2) Vestibular nucleus → Pedunculopontine tegmental nucleus (PPTN) → supramammillary nucleus SUM → Medial septum → Hippocampus (3) Vestibular nucleus → Thalamus → Parietal cortex → Entorhinal/Perirhinal cortices → Hippocampus [22].	Otopetrin1, Alpha 2 adrenergic receptors [23], Glutamate receptor expression [24], c-FOS, vestibular hair cells [25], cerebellar nodulus of adult rats [26,27,28], TEM of synaptic ribbons [29,30,31,32,33]	Nausea related—cardiac sensitivity to baroreceptor reflex; raised Heart rate; raised cortisol; reduced dominant power on EGG baseline, questionnaire [34,35], Serum: NSE and S100β [36], Otolin-1 [37]. vibration-induced nystagmus [38]	(1) Effects of stress on vestibular compensation and adaptation. (2) Social stress, performance anxiety, other psychological stress—will it impede recovery? (3) Stress impedes motor learning in mice (Fragile X mice).
**Gaze**	1. Gaze Holding/Gaze stability 2. Eye-head coordination 3. Redirecting gaze	Gaze Holding	Test in higher animals: NHP	Visual pathway, Frontal eye fields, vestibular nuclei, cerebellum, oculomotor system, parietal cortex, postcentral gyrus, Entorhinal cortex neurons	Horizontal vestibular-generated eye movement: Horizontal semicircular canal → Vestibular nucleus (Vestibular ganglion) and cerebral cortex inputs (frontal eye field) → Paramedian pontine reticular formation (PPRF or gaze center) → Medial longitudinal fasciculus (MLF) → ipsilateral lateral rectus muscle (eye) and contralateral medial rectus muscle (eye) [39].			Structural changes in cerebellum (conventional and mass-spec imaging), Diplopia, Blurring of vision, vestibulo-ocular reflex. Gaze holding/stability and ability to redirect the gaze with accuracy—integrative Biomarker	
**Locomotion**	1. Tandem Walking (=Beam Walking in Animal); 2. Perturbation during walking 3. Navigating obstacle course while walking (eg. Functional Mobility Test) 4. Statistical modeling of actigraphy data	1. Rotarod 2. Beam walking (=tandem walking); 3. Actigraphy in animals; 4. Open field Test directly in humans when possible.	Animal model tests should be developed: a. DigiGait 2.0 Analysis with perturbation, belt or surface perturbation (=human perturbation during walking); b. Dual task test (Catwalk); c. Rodent obstacle course (=FMT)	Mesencephalic locomotor region (MLR) in the midbrain	(1) Reticulospinal pathway: Motor cortex → Basal ganglia → Mesencephalic locomotor region → Pons/Medulla (Reticulospinal cells) → Spinal cord/Central pattern generator → Muscle [40]. (2) Vestibulospinal pathway	(1) Reticulospinal pathway (major pathway for initiating locomotion): Motor cortex → Basal ganglia → Thalamus → Mesencephalic locomotor region → Pons/Medulla → Spinal cord/Central pattern generator network → Muscle (2) Vestibulospinal pathway (3) Rubriospinal pathway [41]		Behavioral tests. Locomotion and gait as a biomarker associated with NDs	(1) Can be nested in vestibular, posture, and gait construct (2) Static vs. Dynamic postural control is important
**Postural control, Balance**	1. CDP. 2. Get up From Fall Test 3. Induced stepping (hold and release) 4. Body sway test (non-parallel two-leg model). 5. Engaged leg model of body sway (uneven weight distribution)	(1) Rotarod (2) Zebrafish Active Posturography (Zap); (3) Floating Platform Tests–Postural sway–measured by Center of Pressure (COP) Assay (=COP) Test directly in humans when possible.	Animal model tests should be developed: (a) Floating Platform Test (b) Motion Capture Analysis (exists but advanced version can be developed)	Cerebellum, sensorimotor cortex, vestibular cortex, prefrontal cortex	Postural information → Vestibular/Visual/Somatosensory input → Brainstem, cerebellum, thalamus → Temporoparietal cortex (vestibular cortex/posteroparietal cortex) → primary sensory cortex → Supplementary motor area and premotor area (info. integration from hippocampus) → basal ganglia/cerebellum (corticovestibular projections) → Brain stem → Spinal cord (reticulospinal tract) → Muscle [42].	Posture-head stabilization: Inner ear vestibular receptors → vestibular nerve → ipsilateral vestibular nuclei in brain stem → vestibulocerebellum/medial vestibulospinal fasciculus → ipsi/contra projections → motor neurons (neck muscle) Locomotion coordination: Inner ear vestibular receptors → vestibular nerve → ipsilateral vestibular nuclei in brain stem → striatum (thalamic relay)/Lateral vestibulospinal fasciculus → ipsilateral projections → locomotor central pattern generator → motor neurons (trunk and leg muscles) [43].		Rodents: Circling, body sway area, the barycenter, the support surface and the weight distribution of the rats when they were moving or stationary [43].	(1) Operationally relevant. Need to evaluate before EVA (2) Animal models not so useful (2 vs. 4 leg)
**Motion sickness**	1. Graybiel scale (comprehensive) 2. Nausea (0 to 10) 3. Eye strain (0–10)	Not reliable in rodent. Ferrets have vomiting response. squirrel monkey and rhesus monkey—difficult to test		Brain stem and Cerebellum	Input (Visual, Vestibular labyrinth, proprioceptive) → vestibular nuclei → cerebellum → brainstem autonomic centers→ vomiting center [44].			Structural changes in inner ear. Increased plasma glucose [45], Nausea related—cardiac sensitivity to baroreceptor reflex; raised Heart rate; raised cortisol; reduced dominant power on EGG baseline [34,35]	(1) Study the effects of stress, sleep deprivation, head-loading, oscillation vibrations, prolonged fixation, and motion sickness (2) There are enormous differences in individual susceptibility, with respect to both sensitivity and adaptation/rapid decay of stimulus. So, in long term space missions like to Mars- should we pre-screen the astronauts? But predicting susceptibility is unclear. (3) How relevant is it to astronaut performance considering it affects only during g transitions (~1% of their time in a 3 year mission). (4) Sopite syndrome—can affect operational performance—Combined effect. (5) Translatability -ferret and mouse model, tricky to track
**Proprioception**	1. Force and joint position test; 2. Dysmetria (finger to nose) test +/− eyes closed; 3. Foot sensitivity via pressure algometry (provides objective measure) = Von Frey Fibers; 4. Thesiometry, vibration at different frequency ranges for slow or fast adapting sensors 5. Tendon tap test, tonic vibrations? complementing Hoffman reflexes	1. Von Frey Fibers; 2. Static force von Frey 3. Two-choice mechanosensory assay 4. Cotton swab assay 5. Tail Clip assay 6. Tape response assay 7. Hargreaves assay 8. Randall-Selitto assay 9. Complete Freund’s adjuvant with von Frey 10. Bradykinin with von Frey 11. Two temperature choice assay. 12. Thesiometry testing—withdrawal responses 13. Coupling a Y maze in dark and add tape for tactile responses. 14. Barrel reception system 15. Whisker test coupled with NOR	Animal model tests should be developed: a. Force and joint position test; b. No identified animal equivalent of dysmetria	Thalamus, Somatosensory cortex, cerebellum, vestibular cortex, prefrontal cortex, Right putamen, parietal cortex, mouse barrel cortex (homunculus)	Dorsal Column pathway: Proprioceptors → Spinal cord → Nucleus cuneatus (Medulla) → Ventral Posterior lateral nucleus (Thalamus) → primary somatosensory cortex Spinocerebellar pathway (unconscious proprioception): Muscle → Spinal cord → cerebellum	Thalamo-insular pathway [46] Proprioceptive signals from Jaw-closing muscle spindles (JCMSs) → the caudo-ventromedial edge (VPMcvm) of ventral posteromedial thalamic nucleus (VPM) → dorsal part of granular insular cortex rostroventrally adjacent to the rostral most part of the secondary somatosensory cortex (dGIrvs2) Proprioceptive signals → thalamus → cerebral cortex	Piezo2 [47], Erg3 transcript levels [48]. Transient receptors which are responsive to camphor, menthol, and capsaicin to stimulate the receptors and check the response.	fMRI and Diffusion tensor imaging (DTI): structural differences within the right putamen [49]-not done in orbit	(1) Very little data from peripheral nervous system and spinal cord. (2) Need to look at the effects of combined stressors
**Fine motor** **control**	1. Peg board; 2. Fine motor test (Holden iPad); 3. String/rope pull 4. Precision grip post-flight (JL)	1. String pull; 2. Spaghetti eating; 3. Lever manipulation	Animal model tests should be developed: Peg board	Cerebellum, basal ganglia, motor cortex, thalamus, rubrospinal, sensorimotor cortex, prefrontal cortex, frontal lobe	Vestibular/Visual input → Brainstem, cerebellum, thalamus → Temporoparietal cortex (vestibular cortex and posterior parietal cortex) → S1 (Primary sensory cortex) → M1 (Primary motor cortex) → Lateral corticospinal tract → Spinal cord → Muscle [42]	Visual/Olfactory input → Sensorimotor cortex → Corticospinal tract (Motor and Sensory) → Cervical spinal cord → Sensory and Motor neurons → Muscle [50]		Isometric pinch grip force between the thumb and index finger [51]	(1) Proprioception can be connected to the fine motor control. (2) Animals have fine motor control, but we need to standardize and develop a model
**Perception**	1. Depth—Egocentric distance 2. Motion illusions—Verbal reports of illusions when changing modules or looking outside 3. Time—Duration estimates	1. Shape—Novel object recognition 2. Depth—Cognitive Flexibility 3. Time—Navigation and Foraging 4. Visual—Food protection behavior	Test in higher animals: NHP	Time perception: Frontal cortex, basal ganglia, parietal cortex, cerebellum, and hippocampus, lateral and medial entorhinal cortex [52]	Dorsal stream pathway (where): Retina → Visual cortex (V1, V3) → Middle temporal area (V3A/MT/V5) and Medial superior temporal area → Intra-parietal area → Parieto-occipital area (PO/V6) Ventral stream pathway (what): Retina → Visual cortex (V1) → Visual cortex (V2) → Visual cortex (V4) → Inferior-temporal cortex → Fusiform gyrus (Fusiform face area and occipital face area) [53]			Structural changes in somatosensory cortex, Perception as a biomarker?—has many confounding factors	(1) Adaptation following flight + return? (2) Some disagreement regarding the relevance of perception in performing operationally relevant tasks
**Pain**	(1) Back pain (2) Skin sensitivity (3) Pain modulation while modulating vestibular sensitivity (4) Joint pain		Crew after one-year long duration mission had significant skin sensitivity for prolonged periods	Thalamus, Primary somatosensory cortex	Pain or Nociception Pathway: Ascending: Nociceptors in Skin → Spinal cord → medulla → midbrain → Thalamus → Primary somatosensory cortex. Descending: Amygdala → Hypothalamus → PAG → rostral ventromedial medulla → spinal cord → nociceptor [54,55]	Ascending pain pathway: Nociception receptors → spinal cord dorsal horn → parabrachial nucleus (brain stem) → thalamus and amygdala → somatosensory cortex/prefrontal cortex/anterior cingulate [56]	Bilateral lesion in mPFC [57]	Blood: MFAP3, GNG7, CNTN1, LY9, CCDC144B, and GBP1 [58], sICAM-1 [59], fMRI based brain imaging [60], Autonomic nervous system markers: Pupil reflexes, Electrodermal activity, Peripheral pulsatile component of cardiac cycle, Heart rate, Blood pressure [61]. Blood markers, miRNA markers, inflammatory factors and CCR2 receptor, Pain as biomarker (many confounders).	(1) Need to focus on peripheral nervous system and include and utilize blood markers. (2) Individual pain tolerance is variable
**Smell and taste**	1. University of Pennsylvania Smell/Taste identification Test scratch and smell test	1. University of Pennsylvania Smell/Taste identification Test in animals—odor is very important, social interactions, fear conditioning, memory sequences of odor.	Smell and Taste has been hypothesized to be modified secondary to fluid shifts causing increase in salt and spice intake leading to dysregulation of body salt composition	Gustatory and olfactory cortex, Piriform cortex and homology to hippocampus. Olfactory epithelial, like hippocampus, has continual neurogenesis	Gustatory pathway: Tongue → solitary nucleus (medulla) → thalamic nucleus (ventral posterior medial nucleus) → gustatory cortex → hippocampus (identification) Olfactory pathway: Olfactory receptors → olfactory bulb → olfactory cortex → hippocampus (odor memory) Olfactory receptors → olfactory bulb → olfactory cortex → thalamus → orbitofrontal cortex (conscious perception of smell)	Olfactory pathway: Odor input → olfactory sensory neurons in olfactory epithelium → olfactory bulb → hippocampus → amygdala → learning/behavioral input [62] Smell and hippocampal circuits are similar → can be used to assess broader cognitive dysfunction	Olfactory bulb volume [63]	Nasal mucus (smell): Sonic hedgehog levels [64]; Saliva (taste)—Sonic hedgehog [65] Blood—miRNA panel including mitochondrial stress markers. Smell test: Scratch and sniff test. Smell as a biomarker.	(1) Loss of smell impacts social interaction and can lead to depression. Loss of smell in long term missions can contribute to depression. (2) Smell can also have a downstream effect. Onset of smell precedes for many years in AD patients. (3) What about systemic response associated with smell deficits; can we have blood biomarkers for it? Mitochondrial functions are associated with olfactory pathways—can we test mitochondria? can we identify miRNAs associated with olfactory issues?
**Hearing**	1. Otoacoustic emission 2. Auditory evoked potential analysis	1. Otoacoustic emission 2. Auditory evoked potential analysis	Test in higher animals: NHP	Auditory cortex	Auditory pathway: Ear → cochlea → cochlear nucleus (medulla) → superior olive (medulla) → inferior colliculus (midbrain) → medial geniculate (thalamus) → auditory cortex Lemniscal auditory pathway, olivo-cochlear system	Ascending auditory pathway: Ear → Cochlea → Cochlear nucleus → superior olive → inferior colliculus → medial geniculate nucleus (dorsal thalamic nucleus) → auditory cortex [66]		Blood: Prestin [67,68], Low frequency hearing loss	(1) Need to study combinatorial stressors (2) Effects of microgravity on hearing/auditory. (3) Largely ignored—as most of behavior test do not rely on hearing ability

### 3.2. Behavioral Medicine Influences on Operational Performance (Leads: C. Davis, David Dinges)

The goal of Group 2 was to create lists of biomarkers and brain regions and/or neural circuits that are related to operational performance for constructs that are prioritized in the HRP’s Behavioral Medicine (BMed) risk. Group 2 assessed the following key constructs which are summarized below and in Table 2: memory, attention and dual tasking, executive function, working memory, learning and plasticity, social processes, individual behavioral states, arousal and regulatory, emotional regulation, risk taking/tolerance, and stress.

#### 3.2.1. Summary of Discussions

Many of the themes that arose during this panel’s discussion were also discussed by the sensorimotor group (Group 1), including learning and plasticity for assessing an astronaut’s general level of adaptability. The panel also discussed the importance of studying individual differences in these different behaviors, in addition to various modifying factors, such as sex, age, the impact of stress, and immune status. The panel also highlighted the importance of general biomarkers that are not specific to any construct, behavior, or tissue, but could provide a more accurate reflection of overall behavioral health.

Behavior is a biomarker. One major theme that emerged from the discussion was the fact that behavior is an important biomarker. Although biomarkers and brain regions and neural circuits are important for understanding the biological basis of changes in operational performance, the behavior itself needs to be studied as an indicator of changes in operational performance. Variations in behavior, such as increases in variability of response and instability in performance, are often the most sensitive indicators of degradation of operational performance [69,70]. Furthermore, marked inter-individual differences exist in these domains, some of which appear to be phenotypic [70,71]. However, limited knowledge exists regarding the biological basis of these individual differences and how they are modulated by spaceflight stressors. For several constructs, the panel noted specific behavioral changes that should be considered as biomarkers and gave examples of potential neuroimaging modalities that could be used to investigate underlying brain regions and neural circuits. More studies of human behavior in spaceflight are needed. Behavioral tests with greater ethological relevance to animal models would most likely yield better translation of findings to human operational performance. The panel discussed similarities between attention tasks and dual tasking; performance instability, increases in the variability of responding, and increased impulsivity are all behavior markers indicating a problem [70,72,73]. These changes can be subtle, which highlights the importance of knowing the organism’s baseline performance for a task, so that changes to that baseline will then indicate a problem. Finally, behavioral biomarkers can be used to determine when an organism—from rodents to humans—is unable to use new information in the environment to adapt their behavior; these results have been obtained primarily from reversal learning and extinction tasks that are highlighted under General Brain Plasticity below.

Common measurements for studying brain biomarkers. Various neuroimaging modalities were discussed for most of the constructs, and because the panel focused on measures that could be assessed during spaceflight and across species, electroencephalogram (EEG) and event-related potentials were regarded as valuable for identifying markers associated with several constructs, including memory, working memory, attention, dual tasking, and learning and plasticity. The use of whole-brain and region-specific EEGs were both considered useful, with whole-brain EEG being particularly important for learning and plasticity [74,75]. Region-specific EEGs were regarded as most useful when coupled with a behavioral task dependent on that region, such as frontal cortex activity and attention or performance on an adaptive N-back test to assess working memory. Near-infrared spectroscopy (NIRS) and functional NIRS were also regarded as useful for assessing underlying neural targets during task performance during spaceflight.

Magnetic resonance electroencephalography and other frameworks for integrating multiple imaging modalities should also be investigated, such as joint imaging markers from simultaneous magnetic resonance imaging (MRI) and EEG (e.g., temporal volume, cortical thickness) that are associated with cognitive status in healthy individuals, pathophysiological changes in neurodegenerative diseases, and after traumatic brain injury [76,77,78,79,80,81]. The panel contended that these simultaneous recordings could provide a more accurate diagnosis of pathology than either modality alone.

Overlapping markers among constructs. The panel agreed that many biomarkers overlap among the constructs, such as the gastrointestinal (GI) microbiome, immune markers, and the influence of steroid hormones. As such, these markers could be general markers of behavioral health. For translational studies, most of these markers can be measured in animal models and have supporting preclinical evidence to demonstrate their relevance to human CNS function and disease.

- Immune markers. Several accessible biomarkers are common to various constructs, including inflammatory markers such as Tumor Necrosis Factor alpha (TNF-alpha), Interleukin 6 (IL-6), and Interleukin 8 (IL-8).
- Oxidative stress markers. The panel considered transthyretin (TTR) as a biomarker of neuronal stress that could be useful for assessing general CNS health, irrespective of a specific BMed construct. Although TTR is possibly inaccessible for spaceflight (e.g., choroid plexus TTR, lumbar puncture for cerebrospinal fluid), recent work suggests serum levels could be indicative of CNS pathology [82].
- Microbiome. The GI microbiome is connected to the brain through the gut–brain axis and the panel regarded this as an important system to assess potential biomarkers indicative of CNS pathology. Recent research demonstrates a vital role of the GI microbiome in CNS pathology and psychiatric disorders [83,84,85] and the microbiome has important implications for health during long-duration spaceflight [86,87].

Incorporate modifying factors into biomarker studies. The panel discussed additional factors important for spaceflight, and differences in many of the BMed constructs that were not included on the worksheet, such as sex, age, stress, immune status, steroid hormone levels, and prior experiences. The panel noted that any findings regarding the usefulness of the various biomarkers should also include tests of these biomarkers under these additional conditions to determine if the markers were relevant when these other factors are included. For example, a biomarker might be useful for males, but not females, or the menstrual cycle phase could impact the usefulness of the biomarker in females. Studying biomarkers under combined spaceflight factors in analog environments [88] was also viewed as being important to determine the usefulness of these biomarkers, given that individuals might respond differently to various spaceflight factors.

Default mode network (DMN). The panel discussed the importance of the DMN in both normal and pathophysiological processes as it relates to several of the BMed constructs, and they considered DMN to be a marker that might overlap among constructs (e.g., changes in DMN could indicate memory and attention problems, in addition to sensorimotor changes). The DMN is a brain system that is preferentially activated when the brain is at wakeful rest [89,90]. Core regions of the DMN include the medial prefrontal cortex, posterior cingulate cortex, and parts of the precuneus, as well as the hippocampus, retrosplenial cortex, and angular gyrus [91]. Changes in activation of the DMN have been associated with several psychiatric conditions, including post-traumatic stress disorder, Alzheimer’s disease, autism, depression, and chronic pain [92,93,94,95,96]. DMN activation can be modulated by different interventions and physiological processes, including physical activity and exercise, sleeping, resting wakefulness, sleep deprivation [97,98,99], and age [100]. The panel regarded the DMN as an important biomarker of brain function, and given its relationship to other cognitive functions (e.g., attention), they thought it could be useful for understanding changes in operational performance. Because the DMN could be an important marker associated with multiple constructs (e.g., memory, working memory), the panel suggested it could also be an important marker for integration of these constructs and/or how modifying factors influence these constructs (e.g., sleep/wake and sleep deprivation). The DMN seems to be essential to the social understanding of others and could provide a biomarker for spaceflight-associated changes in social cognition and behavior.

#### 3.2.2. Recommendations

The panel evaluated each specific construct to determine if a good biomarker exists that is operationally relevant for astronauts and that translates from animal models. The panel also commented on gaps in each construct that would need to be filled to produce an effective biomarker.

1. Attention. The panel identified several important behavioral markers from attention tests, primarily the psychomotor vigilance test, including increased variability in responses, decreased psychomotor speed, impulsivity, instability in performance, and lapses of attention. Several of these performance measures have been studied on the International Space Station (ISS) and in various analogs of the spaceflight environment [101,102].

2. Dual tasking. This construct overlaps BMed and sensorimotor effects and demonstrates the interconnectedness of numerous constructs relevant to operational performance. Furthermore, dual tasking is argued to be a useful behavioral method for assessing changes in cognitive reserve [103,104,105] during spaceflight and after g-transitions after landing [72,73]. Dual tasking measurements during long-duration spaceflight have identified long-term deficits in visuomotor performance and that cognitive reserve is reduced, possibly due to continued sensorimotor adaptation and stress [72]. Dual tasking measures could be useful behavioral biomarkers of how individuals adapt to the spaceflight environment.

3. Procedural memory**.** This form of memory [106] was not specifically identified in the two different memory constructs, but the panel felt that it is essential for operational performance and should be mentioned as a subheading under the memory construct.

4. General brain plasticity as an important biomarker of adaptability or lack of adaptability. Operational performance requires a brain that can adapt to stressors under various spaceflight conditions. As such, alterations in brain “adaptability” could be a useful biomarker indicating degradation in operational performance [107]. For example, simple adaptation to repetitive stimuli or general adaptation across multiple tasks (not only task-specific changes) might indicate how the nervous system is faring in a space-like environment (i.e., whether the brain is able to adapt to this new environment, and whether this adaptability is changing over time). This construct is important because it integrates across all measures, can be translated between rodents and humans, and clinical markers of brain damage exist that could be useful biomarkers (e.g., blood brain-derived neurotrophic factor [88]). In addition, learning and plasticity are constructs that have been tested in animal models relevant to astronaut performance (e.g., reversal learning, extinction learning), including after space radiation exposure [108,109].

5. Reversal learning is used extensively in animal models to assess cognitive flexibility and translates well between rodents and humans [110,111]. The panel suggested that reversal learning under stress or under multiple spaceflight stressors could be paired with neuroimaging (e.g., EEG) to identify factors that impair brain adaptability, and to allow translation from rodents to humans.

6. Although social processes were listed as a standalone construct, the panel noted that social interactions are important for the other constructs, and can be affected by the way individuals interact, the way the crew interacts, and how they perceive the interactions of others or the emotional states of others. This is not trivial and is not necessarily easy to assess, but it is integrated into all other constructs. These interactions highlight the need to consider how these individual states impact the group, and the need to determine if there are biomarkers of these interactions, and/or if those interactions then change the individual biomarkers.

7. Inclusion of additional constructs. When the panel took a broad view of the worksheet, they concluded that additional constructs should be added. Although many of these additional constructs were embodied within some of the other constructs, the panel thought they should be discussed as discrete constructs and how they affect operational performance.

Emotion regulation. This includes dysregulation that is subclinical, but not psychiatric disorders such as depression or anxiety, because those are included in the individual behavioral states construct.

Executive function. Assays to measure executive function were included in the attention construct, but executive function, irrespective of attention, is important to operational performance.

Risk taking/tolerance. The Balloon Analog Risk task is included within the astronauts’ Cognition Test Battery test, and the panel thought that risk taking/tolerance should be a discrete construct and not embedded within another construct. Risk taking/tolerance is also important for social interactions and group dynamics [112] and should be examined in animal models under different spaceflight stressors.

Stress. For example, astronauts’ self-reported stress ratings increased during 6-month ISS missions [102,113] and these changes could have important implications for the usefulness of biomarkers throughout the mission.

The panel identified the following gaps in knowledge:

Lack of integrated approach. The panel noted several gaps that could be addressed by first taking an integrated approach to these different constructs. For example, sleep loss or stress will most likely affect all constructs on the list. The constructs are intertwined, and many things can affect them, and for this reason, our group suggested the use of more general biomarkers, instead of construct-specific biomarkers; for example, a “general health” biomarker or a “vulnerability” biomarker that would indicate an individual’s status on some continuum of functioning within the spaceflight environment. What remains unknown is whether the biomarkers that have been identified are informative under all conditions, or if these markers will change as external stressors and internal conditions change.

Importance of stress. The panel noted several modifying factors, but stress emerged as a critical factor that probably deserves its own category on the worksheet.

Lack of sex differences or inclusion of sex. Sex needs to be considered throughout all the constructs. It was not included in any construct and could have important implications for determining what biomarkers are relevant and useful.

Inclusion of microbiome. This appears to be important to brain function, and as such, could affect the majority of the BMed constructs. A better understanding of the specific bacteria, dysbiosis, etc., and how they relate to cognition and the different performance constructs, would be useful for biomarker development.

Lack of measurements for individual differences. The panel noted the importance of inter-individual differences for these constructs and their likelihood of affecting operational performance. All individuals can be trained with the same techniques, but it is not known, nor can we currently predict, how each individual will continue to perform in the spaceflight environment. This is especially true when hazards such as radiation exposure and isolation are combined. Methods are required to measure these differences and to understand how they might impact operational performance.

Additional gaps. These include the need for better technology to quantify biomarkers during spaceflight, and greater understanding of the differences between diurnal humans and nocturnal animal models (e.g., rodents) and how this influences the biomarkers we identify and study.

**Table 2:** Circuits and biomarkers for behavioral medicine domains.

Key Indicator/Construct	Human Performance Test	Animal Performance Test	Caveats/Notes/Related Functional Performance Tasks/Prediction of Behavioral Outcome in Humans	Brain Region	Human/NHP Neural Circuit/Pathways	Rodent Neural Circuit/Pathway	Biomarkers (Rodents/Humans/NHPs)		Gaps/Notes
							Inaccessible	Accessible (Translatable to Astronauts)	
**Memory**	Mnemonic similarity test (MST) (BPSO)- this test includes Novel object recognition (NOR)	1. Object in place 2. Social Recognition 3. Novel Object Recognition 4. Morris Water maze 5. Fear conditioning 6. Temporal Order 7. Mnemonic similarity test (MST) (BPSO) 8. Barnes Maze	- Needed for recall of training, what you did minutes, hours, days ago - Age-related cognitive decline; mild cognitive decline (MCI); neurodegenerative conditions and dementia - Post-trauma or prior memory testing administration of glucose to activate hippocampus and contextual learning	Hippocampus and associated regions	Excitatory trisynaptic circuit Direct memory formation: Entorhinal cortex → Dentate gyrus → CA3 → CA1→ Entorhinal cortex V Indirect episodic memory retrieval: Entorhinal cortex → Dentate gyrus → CA3 → CA1→ Subiculum → Entorhinal cortex [114,115]	Excitatory trisynaptic circuit	CSF: APOE, amyloid. Hippocampus: decreased BDNF, increased GFAP, inflammatory marker, synaptic marker, Arc	Imaging-CT, fMRI, PET, EEG, MEG, TMS scan for Default mode network activity, mismatch negative amplitude, hippocampal sharp wave ripples (rodents), no contrast fMRI for glymphatic system. Blood: APOE, amyloid, TREM levels, d-cycloserine, neurofilament light chain, BBB breakdown. Behavior -fMRI, EEG and ERPs with behavioral test and stressor. GI microbiome. NIRS/fNIRS	(1) Study effects of Stress, immune system? (2) Study the effect of Combined stressors? (3) Sex Differences? (4) Resource constraints for spaceflight mission—development of readily accessible and implementable technology for biomarker quantification (5) Ethologically relevant animal tests that are relevant to human performance tests
**Attention and dual tasking**	1. Reaction time- PVT 2. Dual Task Test (e.g., cognitive-motor, divided attention): a. PVT b. Walking with distractors 3. Odd-ball stimulus	1. PVT 2. Attention set-shifting: 3. 5C-CPT 5 choice continuous performance test (selective attention)	Used operationally as go/no-go test; operational activities requiring high skill might get most affected; PVT should be considered for performance under pressure with distractions	Prefrontal cortex (lateral PFC) and anterior cingulate cortex	Selective attention: Visual cortex → Lateral intraparietal cortex or Middle intraparietal sulcus → prefrontal cortex [116,117]	sustained attention (PVT/CPT): pedunculopontine tegmental nucleus (PPTN) → substantia nigra pars compacta (SNc) → striatum and PFC → motor control (cholinergic output) [117]	Catecholamine—Noradrenaline, dopamine, mAChR and nAChR	Imaging: fMRI, PET, EEG scan [118,119], EEG of frontal cortex with behavioral task, pupil diameter, NIRS/fNIRS; Urine: norepinephrine, 3-methoxy-4-hydroxyphenylglycol; Plasma: monoamine oxidase, neuropeptide Y [120], Zinc, ferritin; Saliva: cortisol, Genetic and behavioral biomarkers, inflammation related systemic markers. Behavioral markers—Increase in variability of response, impulsivity, instability in performance, attention lapses, dual tasking (motor control + primary task). ECG heart rate measurement, autonomic measurements, and rest activity cycles with task performance GI microbiome; polysomnography (in sleep) and skin conductance/EDA	(1) Correlation between attention, stress, immune dysfunction, and sleep. (2) Predictive validity of operational performance in astronauts—No data on that. Also need rodent and human analogs. (3) Access to operational task data and self-monitoring data (4) Wearable devices for continuous monitoring of heart rate, sleep/wake cycles, rest activity and other autonomic activities without disrupting other crew activities/adding crew time. (5) Continuous and close tracking of crew behavior. (6) Note the bias towards response and response strategy of an individual and its dependency towards individuals’ motivation.
**Working Memory**	1. Fractal 2 back 2. Object rotation in space 3. Spatial WM	1. Radial arm water maze-trials to criterion, latency is common across studies, can be modified for each individual animal, can be modified for test-rests 2. modified Barnes maze (operant n-back in rodents lacks stable baseline) 3. NHP: touchscreen, saccades 4. Elevated plus maze and elevated zero maze 5 Forced swim test 6. Light-dark box without elevation 7. Tail suspension test 8. Puzzle box paradigm—adaptive light/dark box with plugging the hole with various substances (mouse) 9. Unconstrained cognitive flexibility—Novel solutions to the problem (Britten test)	- Docking: Egress procedures and EVA-related;—Crew should stop with plans for completion/performance of task with possible catastrophic consequences if not performed correctly—Anxiolytic effects—Anti-depressive effects—Exploratory behavior and measure of anxiety in open areas	Fronto-parietal brain regions, including the prefrontal, cingulate, and parietal cortices and mediodorsal thalamus (rodent, [121])	Prefrontal cortex → Visual component	PFC-hippocampus (dorsal)—visual component	Rodents-microglia activation in prefrontal cortex and hippocampus, Afg3l1, Tpx2, Neuroligin-3, RB1-inducible coiled-coil 1, Mast3, Kif21a, DnaJ (Hsp40) homolog, SLIT-ROBO Rho GTPase-activating protein 2, Rasgrf1 [20]	Imaging: CT, fMRI, PET, EEG, MEG, TMS scan for default mode network, Neuroimaging with adaptive N-back task, dopaminergic system, whole brain or targeted frontal, parietal, and striatal region Blood: cortisol levels, immune cytokine -chemokine levels (TNFa, IL8, IL-1ra, Tpo, VEGF, CCL2, CCL4, and CXCL5) [122]. Salivary: immune markers. Eye: blink rate for indicator of dopamine sensitivity. GI microbiome, NIRS/fNIRS	(1) Cross-cutting issue with immune markers? (2) Integrative approach
**Learning and** **plasticity**	1. Sequence/procedural; 2. Eye-Head/Eye-Head-Hand adaptation tasks—(a) VOR adaptation test (not that relevant-MS) (b) Eye-Head Hand-visuomotor adaptation task 3. Whole body tasks (a) Walking with visuomotor adaptation (b) Split Belt Locomotion Test 4. Mismatch negativity. 5. Gaze control. 6. Reversal learning	1. Odor sequence learning (non-motor) 2. Eye Head and Eye Head Hand adaptation tasks—(a) Nystagmus and compensation following labyrinthectomy (b) Rodent VOR test 3. Whole body tasks: Ladder rung walk test 4. Mismatch negativity (plasticity + perceptual learning, non-motor component, EEG measure) 5. Barnes maze 6. Extinction learning (Fear extinction). 7. Reversal learning (under stress) 8. Delayed matching to position (DMP) 9. Radial arm maze	- Adaptability is an important trait that will need to be tested with combined stressor because of the need to adapt rapidly after g transitions	PFC, hippocampus (depending on test), cerebellum, striatum (depending on motor component of the test), sensorimotor cortex	Trisynaptic pathway, working memory circuitry	Trisynaptic pathway	ARC, cFos, synaptic markers, BDNF, MMP-9 levels, microstructure of constrained motor connectome and corticospinal tract [123]	CT, fMRI, PET, EEG, MEG, TMS scan. EEG of whole brain for plasticity and adaptation with task or repetitive stimuli. Blood- BDNF. GI microbiome. NIRS/fNIRS	Convergent tests-adaptable to operational tasks
**Social Processes (e.g.,** **Socialization,** **conflict, communication, bonding)**	Socialization: Self-report survey, sociometric badge Conflict: Self-report survey, journal analysis, observational ratings Communication: Self-report survey, communication recording analysis, observational ratings Bonding: Observational ratings	Socialization: 1. Social fear 2. Social approach to a stranger mouse 3. Reciprocal social interactions 4. Conditioned place preference to conspecifics 5. Social recognition 6. Juvenile play 7. Nesting patterns in home cage Conflict (Aggression) 1. Social Defeat 2. Resident intruder attack 3. Routine observation 4. Isolation-induced fighting 5. Tube test for social dominance Communication 1. Ultrasonic Vocalizations emitted during social interactions 2. Response to vocalizations form conspecifics 3. Deposition of social olfactory pheromones Bonding 1. Pair Bonding 2. Observation, Grooming, Inter/Intra-Social Interactions 3. Oxytocin/Vasopressin levels Social Hierachy 1. Hierarchal testing/Social stability measurements—convergent testing like tube testing 2. Urine marking (sex should be considered) 3. Hotspot testing		Prefrontal cortex, Amygdala, Hypothalamus, striatum	Aggression: Sensory reception → Prefrontal Cortex → Amygdala → Hypothalamus → Periaqueductal grey (midbrain)/Ventral Tegmental area → Aggressive behavior [124]	Social attachment: Olfactory cues → Vomeronasal organ (VNO)/Main olfactory epithelium (MOE) → Accessory olfactory bulb (AOB) → Amygdala → Lateral Septum→ mPFC → Nucleus accumbens Dominance: (1) Olfactory cues → VNO/MOE → AOB → Amygdala (2) Social stimuli → mPFC → Nucleus accumbens/Hypothalamus/Amygdala/Ventral tegmental area./Dorsal raphe nucleus/hippocampus Aggression: Olfactory cues → VNO/MOE → AOB/Main olfactory bulb (MOB) → Amygdala → Hypothalamus/Bed nucleus of the stria terminalis (BNST)/Hippocampus (Hippocampus→ Lateral Septum) [125]	TRPc ko mice (loss of aggression) [126], reduced/loss of nNOS (increased aggression and reduced social investigation) [127], Neuroligin-3, PSD95, parvalbumin, bone hormone-osteocalcin. Radiation studies in brain—CCL2, CD206, CD163, PSD-95 in PFC, Dopamine receptor levels	CT, fMRI, PET, EEG scan Blood-Vasopressin and oxytocin levels, 5-HT, nNOS (male mice), testosterone (social regulation), cortisol, progesterone, cortisol to testosterone ratio, cortisol to oxytocin ratio. Imaging- Striatum and reward related brain regions. Psycho variables—heart rate, skin sensitivity. GI microbiome; NIRS/fNIRS; polysomnography (in sleep) and skin conductance/EDA. Behavior-eye gaze and eye tracking	(1) Learning effects and sex difference (2) Behavior of one animal/an astronaut would affect others behavior (3) Understanding the dynamic social interaction between the crew members, psychological ownership of the space, habitat size to social interaction and any areas that need mitigation.
**Individual Behavioral States (e.g., Stress,** **Depression, Mood and** **Anxiety)**	Stress: Visual Analog Scale Depression: Beck Depression Inventory Mood: Profile of mood states-short form, Zung self-rating depression scale, Hamilton Rating Scale for Anxiety, Beck Scale for suicide Ideation and Beck Hopelessness Scale, Quality of Life Enjoyment & Satisfaction Questionnaire, Psychological General Well-Being Index, Pittsburgh Sleep Quality Index Risk Tolerance: balloon analog task	Stress 1. Immobilization Depression 1. Forced swim test 2. Inescapable shock 3. Low sucrose preference (Anhedonia) 4. Tail suspension 5. Social defeat 6. Leaned helplessness 7. Novelty-Suppressed Feeding Mood 1. High elevated plus maze 2. High changing reinforcement schedules 3. High open field avoidance Anxiety 1. Light-dark exploration 2. Vogel conflict test 3. Marble buying 4. Unpredictable chronic mild stress Risk Tolerance 1. Elevated plus maze (head dips), 2. delayed reward task (impulsivity), 3. Rat gambling task. 4. Predator odor risk taking test		Prefrontal cortex (PFC), subgenual cingulate cortex (Cg25), subcortical hippocampus, nucleus accumbens, amygdala, ventral tegmental area	5HTergic/NEergic Depression pathway: Locus coeruleus/Dorsal raphe → Amygdala/Hippocampus/Ventral tegmental area/Nucleus accumbens → Prefrontal cortex [128]	5HTergic/NEergic Depression pathway: Locus coeruleus/Dorsal raphe → Amygdala/Hippocampus/Ventral tegmental area/Nucleus accumbens → Prefrontal cortex [128]	Choroidal plexus: TTR (independent of radiation exposure). CSF: Glutamate, GABA, Acetylcholine, Norepinephrine, Dopamine, Serotonin, Vasopressin, Orexin. Tissue: MAPT, HTT, Presenelin-1, APP (independent of radiation exposure), glial and synaptic dysfunction	fMRI scan Blood: Glutamate, GABA, Acetylcholine, Norepinephrine, Dopamine, Serotonin, Vasopressin, Orexin, cortisol, corticosterone, Immune markers: IL6, B-cells, Cortisol, TNFa, IL4, IL5, IL-10 [122,129,130], CSF—TTR (lumbar puncture) Saliva: Cortisol; NIRS/fNIRS.	(1) How individual behavioral state will impact the others in the group (cohesion, behavioral state of the group). This relates to where the crew is in the craft and who interacts with whom, crew member who isolates themselves can be a behavior issue to be detected and dealt with.
**Arousal and Regulatory (e.g., sleep, circadian phase)**	Sleep duration and Architecture: Actigraphy and EEG 1. PVT 2. Visual analog scale towards alertness—assessing sleep quality Circadian phase: Actigraphy (not good biomarker)	Sleep duration and Architecture: Actigraphy, Sleep Island, EEG Circadian phase: Actigraphy (not a good biomarker)	Sleep duration and Architecture: Actigraphy and EEG, PVT, sleep quality Circadian phase: Actigraphy (not good biomarker)	Hypothalamus, Brain stem, Spinal cord, Suprachiasmatic nucleus	Sleep: Retina (light) and metabolic inputs (peptidergic hormones, nutrient signals) → Retinohypothalamic tract and Arcuate nucleus → suprachiasmatic nucleus → ventral sub paraventricular zone → dorsomedial hypothalamus → ventrolateral preoptic nucleus → sleep Wakefulness: Retina (light) and metabolic inputs (peptidergic hormones, nutrient signals) → Retinohypothalamic tract and Arcuate nucleus → suprachiasmatic nucleus → lateral hypothalamic area (melanocyte concentrating hormone/orexin-producing neurons) → wakefulness [131]	Circadian rhythm: Retina → Retinohypothalamic tract → Suprachiasmatic nucleus → Paraventricular nucleus → Medial forebrain bundle → Intermediolateral cell column → Superior cervical ganglion → Nervi conarii → Pineal gland (Melanocyte—Melanin secretion) [132]	Brain Melatonin levels (not accurate with rodents) nocturnal animals and light cycle and when the test is conducted (light or dark cycle) Sex difference	CT, fMRI, PET, EEG, polysomnography scan 6-sulphatoxymelatonin (aMT6) collected every 2 to 8 h. over 24 to 48 h period, melatonin, Timeless, period 1–3, growth hormone (SOCS) [133] Actiwatch (sleep quality, duration), Urine: 6-sulphatoxymelatonin (good biomarker); Melatonin in blood and saliva (not accurate), core body temperature (susceptible to masking), GI microbiome, genotype changes—per3 polymorphisms (human), Dqb10602 gene (narcolepsy), Immune markers—IL6; behavioral tests; NIRS/fNIRS	(1) Sex differences (2) Associations between menstrual cycle phase, sleep need and circadian (major gap!) → actually, not only estrogen, but testosterone cycles too, so should consider both! (3) Differences between nocturnal and diurnal species! Most rodents are nocturnal, but most behavioral tests on rodents (in general, not sleep specific) are done in light. (4) New technology for measuring fluid shift and shift of brain in the cranial compartments. Tympanic membrane movement measurement (5) Sleep duration, quality, and continuity. Need to ensure that sleep is: not disturbed, adequate for operations, at appropriate circadian time, entrained by light, exercise etc. Sleep quality is an orthogonal component to stress and emotional status. (6) Diet and its contribution (7) Intersubject variability
**Emotional regulation**				Hippocampus, striatum, PFC					Psychology, subclinical—Facial expression, emotional regulation. Regulation of the conflict. Executive functions.

### 3.3. Integrated Biomarker and Signaling-Pathway Approaches for Understanding Operational Performance (Leads: X.W. Mao, R. I. Desai)

The goal Group 3 was to use a systems-biology approach to generate lists of biomarkers and signaling pathways related to CNS circuitry and operational performance that will be important to monitor in astronauts during spaceflight and after return to Earth. To achieve this goal, the integrated approaches team (a) reviewed and identified a broad array of biomarkers of important mechanisms known from space research (i.e., what is known); this panel discussed research on biomarkers and signaling pathways in animals and humans that could be used to assess the effects of acute or long-duration exposure to spaceflight stressors on operationally relevant performance; (b) considered knowledge from other CNS-health studies that could be repurposed for assessing astronauts (e.g., aging, disorder, disease); and (c) documented open questions and research gaps in the knowledge base that connect genes and biological pathways to brain regions and neural circuits that link to operational performance (i.e., what is not known, needed experiments). Discussions are summarized below and in Table 3 and Table 4. The goal of this integrated approaches team was to provide recommendations regarding the availability, validity, and limitations of biomarkers and signaling pathways to be examined in future research.

#### 3.3.1. Summary of Discussions

It should be emphasized at the outset that the results of this integrated approaches exercise did not reveal any biomarker (or combination thereof) that was uniformly responsive across different regions of the brain to a single or given combination of spaceflight stressors. The panel raised the following distinct, yet overlapping questions:

1. Does the literature provide any useful insight regarding if or how combined exposure to spaceflight stressors might interact to alter (additive, synergize, diminish) biomarkers and signaling pathways involved in CNS function?
2. What experiments need to be performed to inform how these combined stressors interact and affect biomarkers and signaling pathways associated with CNS function?
3. What are the challenges that need to be addressed for data collection and storage?
4. What information do we need for successful biomarker repurposing?
5. What new experiments, analysis, and techniques are needed?
6. What information about biomarkers and signaling pathways is needed to identify and implement effective spaceflight countermeasures that will minimize CNS decrements associated with the long-duration spaceflight beyond Earth’s protective magnetosphere?

Below is a summary of the key issues that were raised by the integrated approaches panel.

1. First and foremost, all group members recognized the need for standardizing certain aspects of the experimental protocol across laboratories; in particular, standardizing (a) factors related to the degree of exposure to a spaceflight stressor (e.g., space radiation (Galactic Cosmic Radiation simulation), dose, dose rate, and energy; isolation/confinement; altered gravitational levels (Mars, lunar or Earth)); (b) the type of animal models used (e.g., age, sex, strain, species; see below) and the time of tissue collection. This approach will permit meaningful comparisons and interpretations of data from different endpoints collected among investigators.
2. The panel overwhelmingly agreed that a paucity of information exists on how CNS-related neurocognitive performance is affected in laboratory animals that have been exposed to space-relevant radiation (e.g., a low-dose (<0.5Gy)/low-dose-rate of simulated galactic cosmic rays) and that such effects have not yet been systematically studied.
3. Although studies using several species (e.g., rats, mice) have provided important information about how spaceflight stressors may affect behavior and cognitive function, extrapolating data from rodents to humans is an imperfect science. Notably, the translational value of larger size animals (e.g., NHPs) used in various research domains, including neurobiological, neurobehavioral, and complex cognitive processes, has been validated and established over many decades. These successes are based on numerous factors including (1) the considerable overlap in the genetic, physiological, pharmacokinetic, neurobiology, and neurobehavioral effects in NHPs and humans; (2) the proven reliability of NHPs as subjects in long-duration (i.e., longitudinal) neurobehavioral and cognitive studies; and (3) the ability to use powerful within-subject designs that are similar to those used in human studies, which permit meaningful conclusions or inferences by evaluating all treatment effects in individuals as well as in groups. Considerations such as these suggest that NHPs are especially well-suited for ground-based study of the acute and long-term neurobehavioral effects induced by spaceflight stressors, either alone or in combination, and for translating effects to astronauts. Thus, there was considerable appreciation in the group that the use of appropriate animal models, especially targeted studies in NHPs to confirm or advance observations in rodents, should be carefully considered by NASA in future work.
4. The panel recognized that an integrated “omics” profiling strategy using technologies such as genomics, proteomics, and metabolomics is desperately needed to further expand understanding of the underlying brain systems/mechanisms that may be affected by exposure to spaceflight stressors. This multimodal approach will be highly beneficial to determine biomarker datasets of differentially expressed genes, proteins, or metabolomic/lipidomic signatures and the pathways that lead to pathological and possible degenerative changes in the brain. An omics-based molecular phenotyping approach for characterizing biosignatures associated with low-dose space radiation, simulated microgravity, and other space environmental stressors will provide a deeper understanding of the underlying mechanisms responsible for brain structure and pathophysiological changes. This approach will also provide critical information about how individual sensitivity (e.g., genetic, epigenetic, previous injury, age, and sex/gender) will influence how spaceflight stressors affect operational performance. However, as stated above, it will be critical for protocols and metadata from experiments in different laboratories to be standardized and processed on a uniform pipeline.
5. A need was identified for longitudinal studies that provide information about changes within the brain (i.e., acute to chronic). This is especially germane for determining if exposure to spaceflight stressors produces short- or long-term neurobiological (or degenerative) adaptations that affect operationally relevant behavioral and neurocognitive performance. A major complication associated with determining how the brain responds to stress insults is the latency between exposure and the expression of injury (e.g., cell loss or dysfunction). Thus, it is essential that longitudinal studies are conducted to meaningfully quantify the development and progression of the CNS injury response.
6. At present, few studies have examined the combined impact of spaceflight stressors on operational performance and/or associated neurobiological changes in the brain. Thus, it is critical that future studies use ground-based animal models that incorporate stressors that are inherent to the spaceflight environment, i.e., space-like radiation exposure and other spaceflight environment stressors including high pCO2, fluid shifts, microgravity, environmental constraints, emotional stress, and circadian misalignment/sleep deprivation. This will permit data to be extrapolated more accurately to estimate potential risks encountered by astronauts during deep space missions. Ground-based studies to examine the impact of combined spaceflight conditions and the underlying mechanism(s) of potential interaction on structural and functional deficits in the brain are very limited.
7. The panel overwhelmingly agreed that significant effort and resources are needed to develop new cutting-edge techniques to identify brain biomarkers that may indicate operationally relevant neurocognitive performance. Novel imaging techniques that provide an early detection of the subtle changes in the brain and identify the target population and biomarkers for intervention are essential. Thus, to improve knowledge about anatomical, physiological, and functional changes to the brain, especially for longitudinal evaluation, an effort is needed to develop advanced computerized tomography scan, functional magnetic resonance imaging (fMRI), positron emission tomography scan, EEG, magnetoencephalography, and transcranial magnetic stimulation scan imaging technologies.

The panel members agreed that a critical need exists to use data better and carefully from flown astronauts to evaluate the actual acute and long-term health risk of the spaceflight environment. Importantly, there was appreciation that human data could be better related to outcomes from animal studies, which may help characterize alterations in circadian rhythm and sleep, immune system, neurotransmitters, neurobiology (i.e., brain structure and function), and vasculature. If used carefully, follow-up analysis of omics, biochemistry, imaging, and a battery of behavior and neurocognitive testing will provide critical human data that may be used to evaluate the actual acute and long-term health risk of the space environment.

#### 3.3.2. Recommendations

Table 3 highlights the major observations and points of discussion that were addressed by the integrated approaches panel. Although it is likely that exposure to combined spaceflight stressors will alter a wide range of biomarkers in different endpoints in animals and humans, ultimately, it is critical that these biomarkers are consistently and reliably linked with changes in operationally relevant behavior and neurocognitive performance. Evidence so far suggests that specific neurocognitive impairments may manifest under evolving mission scenarios (i.e., increased cognitive load) and, therefore, assessing the impact of spaceflight hazards on a wide range of operationally relevant behavioral and neurocognitive tasks is critical. Moreover, the panel suggested that NASA should explore both novel and trained paradigms with increased difficulty of determining the level of impairment. Finally, to promote translation between animal models and humans, parallel behavioral and neurocognitive testing paradigms exist between rodents ↔ NHPs ↔ humans that should be further exploited.

The panel identified the following gaps in knowledge:

- How can data be integrated across many biology scales for CNS endpoints?
- How can system biology approaches with new technologies—organ cultures, organs-on-a-chip made from normal human cells, integrated “omics” (genomics, proteomics, metabolomics) and cutting-edge brain imaging techniques—be used to estimate acute CNS risks to astronauts from space environment?
- How can knowledge of space environment-induced biomarkers/pathways in neuroinflammation, blood–brain barrier function, vasculature, glia activation be integrated towards better understanding of their impact on acute pathophysiological changes in the brain and late neurodegeneration?
- What is the likelihood of increases in the brain susceptibility to later development of neurological disorders as results of observed changes?
- What is the relationship between neurochemical biomarkers and operationally relevant performance?
- What are the temporal and regional differences in neurochemical biomarkers and their influence on operationally relevant performance? What is the right neurochemical balance?
- What CNS neurotransmitter metabolites can be measured peripherally? Can wearable devices/sensors be used instead of blood?
- Is personalized nutrition (i.e., B-vitamin supplementation) a viable SANS countermeasure?
- Do recurring cycles of sleep deprivation affect performance/vestibular/sensorimotor changes, recovery, and biomarkers?
- What is the role of individual susceptibility—genetic, epigenetic, previous injury, age, and sex/gender—in addressing CNS risk?

Information that is lacking includes astronaut data to monitor the level of DNA damage over time; miRNA signatures as neurodegeneration markers for acute/chronic injury; data from integrated phenotypic studies in models; and omics to identify molecular changes at the synaptic level.

## 4. Overall Summary and Recommendations

In total, hundreds of biomarkers have been identified and synthesized through this effort. Synthesizing across all three topical groups, the following common responses emerged as general themes:

- Biomarkers span all levels of data from molecules to behavior.
- Integrated stressors and integrated effects should be studied, including studies using multi-sensory approaches, for example, combined sleep and radiation exposure.
  o Note combined effects of HZE radiation exposure and sleep fragmentation in rodent models show dramatic effects specific to brain regions [109].
  o Integrated sensorimotor and cognition effects should be considered for study, e.g., olfaction and vestibular.
- The responses themselves will have multiple downstream impacts. Treatment may not be successful following a reductionist manner.
- Modifying factors should be identified and tracked throughout assessment, e.g., cognitive load, stress, circadian aspects, and sex, and their impacts on executive function and attention.
- Learning and plasticity were highlighted as critical areas to assess during spaceflight to determine the astronaut’s general level of cognitive and sensorimotor adaptability.
- Biomarkers were recommended not just for immediate predictiveness, but also for long-term predictiveness of damage (late effects that can follow the initial injury by months or longer). As an example, some omics biomarkers may precede pathologies by months.
- Studying appropriate animal models in parallel with astronauts is extremely valuable for determining applicable constructs/responses, and to better understand the astronaut’s condition.

We hope this effort yields usable knowledge and an effective tool for HRP and the CBS Project to improve monitoring and management of astronaut cognitive and behavioral health.

## Figures and Tables

**Table 3:** The major observations and points discussed by the panel.

**Oxidative Stress** Blood biomarkers: 8-oxo-dG in immune cells, MDA, f2-isoprostane, Nitrotryosine; brain HNE, glutathione, lipid peroxidation, ROS, NFKb, MAPK activation, Xanthine oxidase Oxidative stress-associated mitochondrial dysfunction has been shown in many cells, tissue and organ system, their impacts have to be further investigated. The role of diet in mitigating oxidative stress associated with spaceflight. Epigenetic clock measurements in astronauts and related to time in space or deep space and their association with oxidative stress-induced aging. miRNA signatures and exosomes in identifying oxidative stress biomarkers and as novel biomarkers in brain pathogenesis.	**Neurotransmitters** Behavioral biomarkers: mood, depression, anxiety tests Limited to in vitro data that are inconsistent across studies. Only one neurotransmitter examined at a time (e.g., DA, glutamate, 5-HT, or ACh). Human studies with MRI spectroscopy are difficult to do in real-time. Only invasive rodent assays are available. Need studies that associate neurotransmitter changes with changes in lipids/metabolites. Neurotransmitters provide a direct readout of CNS functionality at multiple levels: behavioral, emotional, systemic stress, endocrine, and electrophysiological. Cross-species correlates (chemical changes): rodents– NHP–Humans and should be translated to lipidomic and metabolomic findings.
**Neuroinflammation** Blood biomarkers: COX-2, TREM, IL-4, TNF, BDNF, corticosterone; YKL-40, ICAM-1, VCAM-1, IL-15, and Flt-1 in CSF; Behavioral biomarkers: cognitive tests Specificity of blood biomarkers such as cytokines (variability with circadian changes and time of collection). Animal to human correlation (circadian and sleep system differences, rhythm differences, immune differences, white-matter differences, vasculature differences). Applying cell-free DNA and subsequent methylation analysis can give high sensitivity measurement of BBB integrity, cell breakdown and inflammation in the brain.	**One-carbon metabolism** Blood biomarkers: folate, Vit. B-12, methylmalonic acid and homocysteine, MMPs; CSF: 5MTHF Difficult to correlate biomarker changes between CSF and plasma Genetic variations in folate-mediated one carbon metabolism predict risk of adverse effects in space flight–mechanisms are unknown Relation to white matter hyperintensity, inflammation, BBB permeability, inflammation, behavioral changes, e.g., Hyperhomocysteinemia, vascular dementia.

**Table 4:** Circuits and biomarkers for integrated approaches/physiological responses.

Physiological Responses	Related Gene Ontology Terms	Biomarkers (Human/Rodent)		Associated Pathways/Signaling Cascade	CNS Health Risks	Human Behavioral Measure	Rodent/NHP Behavioral Measure	Open Questions/Gaps (How to Close?)	Notes/Limitations on Biomarkers
		Inaccessible	Accessible						
**Neuroinflammation**	Glial activation, neuron apoptotic process, BBB disruption, endothelial dysfunction, oxidative stress	CSF: YKL-40, ICAM-1, VCAM-1, IL-15, and Flt-1 [134], Brain lysates-CCR2 [135], Brain lysate-proteomics, IHC, IL21. CSF-cytokine (accurate for neuroinflammation)	Blood: COX-2, cytokines, TREM [136], IL4, TNF, BDNF [137], Corticosterone [138], c-reactive protein, IL-6 and TNFa, glial fibrillary acidic protein (GFAP), IL110, IL4 (variability due to circadian disruption or sleep deprivation), IL21 Imaging: CT, fMRI, PET, EEG, MEG, TMS scan, MRS (myoinositol, glutamine to glutamate ratio), Functional biomarker—HSV1 (viral reactivation)	NFKB signaling, Chemokine signaling, TNF signaling, Calcium signaling, Serotonergic synapse, VEGF signaling, Autophagy, oxidative stress	Neurodegenerative disorders, meningitis	Cognition, Mathematical processing (MTH), Running memory continuous performance test (CPT), Delayed matching-to-sample (MTS), Code substitution (CDS)	Spontaneous new home behavior, Elevated plus maze, light/dark box, WMWM and fear conditioning, contextual fear conditioning, Morris water maze test, pass avoidance performance test, climbing pole test	(1) Longitudinal study of blood biomarker (e.g., cytokines) and correlating with individual’s biological clock (variability across individual of approx. 5 h.), clinical and medical history. (2) Flight deployable ELISA cytokine panel (3) Microfluidics based system that can be deployed, miniaturized microscope and flow cytometer. (4) For animal to human study correlation—Tissues can be harvested and animal study should be contextual to the question asked. Humanized mouse model—good for immunological study. (5) Leverage omics data. (6) Countermeasure development requires living system. (7) Other animal model—Canine, pig, marmoset—reinventing the wheel?	(1) Threshold? (2) Challenges for data collection and storage: (3) Unclear whether plasma will be collected and stored in space, then assessed on Earth, or are we looking for measures that can be done in real time in space? Some of these assays require special equipment and assays. Importance of storage consistency-Plasma biomarkers are very sensitive to processing and storage conditions, including type of plastic for tubes, tube size and volume of aliquots. (4) Recommend many small aliquots to maximize potential for number of biomarkers that can be assessed, because freeze-thaw also significant influences measurement. (5) Specificity of blood biomarkers such as cytokines (variability with circadian changes). (6) Animal to human correlation (circadian and sleep system differences, rhythm differences, immune differences, white-matter differences, vasculature differences).
**Neurotransmitters**	Neurotransmitter release and metabolism, cellular metabolism	Brain lysates: Serotonin [139], Dopamine and dopamine regulating enzyme, COMPT [139,140], Ach [139], Norepinephrine, Epinephrine, Glutamate [141], Glutamate receptors (NMDAR2A/2B) [133], Stress hormones-cortisol, oxytocin; Corticotrophin-releasing hormone (CRH); Corticotrophin-releasing factor (CRF) [142]	Blood: Serotonin [139], Dopamine and dopamine regulating enzyme, COMPT [139,140], Ach [139], Norepinephrine, Epinephrine, Glutamate, GABA [141], Glutamate receptors (NMDAR2A/2B) [133], Stress hormones-cortisol Imaging: CT, fMRI, PET, EEG, MEG, TMS scan	Monoamine pathway: mesocorticolimbic; nigrostriatal. Hypothalmic-pituitary-adrenal (HPA) axis	Mood, Depression, Anxiety, Alzheimer’s, schizophrenia, Parkinson’s, other degenerative conditions; Social stress (Stress leading to social dominance)	Mnemonic similarity test (MST) (BPSO)-this test includes Novel object recognition (NOR), learning and motor tasks	Thigmotaxis, water maze, elevated maze, open field test, passive avoidance	(1). What is the relationship between brain neurochemistry and behavior? (2) Are neurochemical signatures differently impacted in different brain regions to influence behavior and what is the right balance? (3) What can be measured peripherally? (4) Which dopamine and serotonin metabolites are best measured peripherally? (5) Wearable devices/sensors to measure metabolites instead of blood tests	Limitations: (1) Inconsistent data across studies: one neurotransmitter system examined (e.g., DA, glutamate, or 5-HT): comprehensive assessment needed. (2) Human studies with MRI spectroscopy are difficult to do in real-time. (3) Rodents’ assays are invasive measures, lack less invasive techniques (4) Need studies that associate neurotransmitter changes with changes in lipids and other metabolites Strengths: (1) Neurotransmitters provide a direct readout of CNS functionality at multiple levels. (2) Cross-species correlated (chemical changes) rodents—NHP—Humans. Should be translated to lipidomic and metabolomic findings.
**One-carbon metabolism**	SANS, BBB, endothelial dysfunction, CSF pressure, Bioenergetics	Brain: B-vitamin and 1C metabolite profiles, DNA strand breaks; uracil in genomic DNA and mitochondrial DNA (higher sensitivity)	Blood: serum and RBC, folate, vitamin B12, methylmalonic acid and homocysteine, MMPs, Met, AdoMet (P. Stover), Formate, one-carbon nutrients, and their methylation profiling (inputs towards one carbon metabolism pathway). Imaging: OCT for SANS, MRI for WMH; skin autofluorescence for AGE; Ultrasound Elastography (scleral stiffness), OCT angiography CSF: 5MTHF	Folate and methionine production, Epigenetic methylation, DNA synthesis and repair, Neurotransmitter metabolism, Trans-sulfuration pathway, Bioenergetic crisis	SANS, Neurodegenerative disorder (AD), neurodevelopment, Depression	Cognition: Standardized Mini-Mental State Examination, simple reaction time (SRT), choice reaction time (CRT), digit vigilance task (DVT)	Cognitive tests (Morris water maze)	Is personalized nutrition (i.e., B-vitamin supplementation) a viable SANS countermeasure?	(1) Correlating biomarker changes between CSF and plasma? (2) Relation to white matter hyperintensity, inflammation, BBB permeability, inflammation, behavioral changes, e.g., Hyperhomocysteinemia, vascular dementia.
**Oxidative stress**	Autophagy, inflammation, Lipid peroxidation, Bioenergetics	Tissue: Glutathione, lipid peroxidation, ROS, NFKb, MAPK activation [143], Blood vessel-Xanthine oxidase [144]	Blood/Urine: Cytokines levels, HNE, MDA, f2-isoprostane, Nitrotryosine levels [145], 8OHdG; reduced/total glutathione, total antioxidant capacity, superoxide dismutase, glutathione peroxidase, advanced glycation end products (AGEs), glycated albumin, 3-nitrotyrosine, oxidized LDL, miR383 (regulating AQP4), cell-free DNA (genetic and epigenetic changes) Imaging: CT, fMRI, PET, EEG scan, PET with 62Cu-ATSM [146]	Oxidative phosphorylation, Mitochondrial dysfunction, NFR2-mediated oxidative stress response, Superoxide radicals’ degradation, Neuroinflammation, apoptosis, necrosis, neurovascular impairments, Bioenergetic crisis	Neurodegenerative disorders, Cardiovascular disorders, affects multiple organs, Anxiety, Depression, Schizophrenia, Metabolic disorders, SANS.	Anxiety and depression related behavioral tests (Visual Analog Scale Depression: Beck Depression Inventory), psychomotor tests (Tandem Walking, Perturbation during walking, navigating obstacle course while walking (e.g., Functional Mobility Test)), Cognitive tests (Mnemonic similarity test (MST) (BPSO)-this test includes Novel object recognition (NOR), Fractal 2B, object rotation in space)	Anxiety related (Elevated plus maze, hole-board, and open field tests), Psychomotor tests (Rod walking, wire suspension/wire hanging, plank walking, inclined screen, accelerating rotarod), Cognitive tests (Morris water maze)	(1) Can diet mitigate oxidative stress associated with space flight? (2) What are the relationships between ox stress, immune function during flight? (3) miRNA signatures? Antagomir-countermeasure, specificity, applicability? (4) Exosomes?	
**Mitochondrial dysfunction**			Plasma: Formate (mito one carbon metabolism) biomarker of mitochondrial function.						
**Synaptic plasticity/Neurotrophic Factors**	Regulation of synaptic plasticity, modulation of chemical synaptic transmission, neurotrophin receptor activity	Brain lysates: BDNF, Neurotrophin-3 [147], synaptophysin [148], CtBP2, Shank1a [29], 14-3-3 proteins (CSF marker of CNS degeneration), EEG markers, BDNF, c-Fos	Imaging: CT, fMRI, PET, EEG, MEG, TMS scan; Plasma: Neurofilament light (NfL), phospho-tau 181 (pTau181), beta-amyloid 40 and 42, BDNF; CSF: NfL, pTau181, beta-amyloid 40 and 42.	Ubiquitin-proteosome, lysophosphatidic acid (LPA), kinases, Calcium signaling (PI3K, PLC gamma), MAPK/ERK	Neurodegenerative disorders, schizophrenia	1.Sequence/procedural; 2. Eye-Head/Eye-Head-Hand adaptation tasks— (a) VOR adaptation test (b) Eye-Head Hand- visuo-motor adaptation task 3. Whole body tasks (a) Walking with visuomotor adaptation (b) Split Belt Locomotion Test 4. Mismatch negativity	1. Odor sequence learning (non-motor) 2. Eye Head and Eye Head Hand adaptation tasks: (a) Nystagmus and compensation following labyrinthectomy (b) Rodent VOR test 3. Whole body tasks (a) Ladder rung walk test 4. Mismatch negativity (plasticity + perceptual learning, non-motor component, EEG measure) 5. Mathematical processing (MTH)	(1) Markers of neurodegeneration are missing. Acute and chronic injury can be tracked longitudinally with plasma NfL. (2) Lacks integration of phenotypic studies in models and omics. (3) miRNA signatures are missing. (4) Identify molecular changes at the synaptic level (5) Relatively unexplored area	Which biomarkers can we repurpose from terrestrial disorders to spaceflight? There have been huge advances in Alzheimer’s and vascular dementia blood-based biomarkers. While associated with aging, these markers can reflect neuronal and vascular injury and later risk of cognitive problems. NfL is a marker of neuronal injury that is increased significantly in traumatic brain injury, many forms of dementia, and CTE.
**Vestibular/Sensorimotor alterations**	Vestibular reflex, vestibular hair cell stereocilium organization, vestibular receptor cell stereocilium organization	Otopetrin1, Alpha 2 adrenergic receptors [23], Glutamate receptor expression [24], c-FOS, vestibular hair cells [25], cerebellar nodulus of adult rats [26,27,28], TEM of synaptic ribbons [29,30,31,32,33,149].	Nausea related—cardiac sensitivity to baroreceptor reflex; raised Heart rate; raised cortisol; reduced dominant power on EGG baseline, questionnaire [34,35], Circadian measurements Imaging: CT, fMRI, PET, EEG, MEG, TMS scan		Motion sickness, Dizziness, Loss of Hearing, Postural imbalance, Vertigo	Cognition, Spatial memory, Graybiel scale, CDP, get up From Fall Test, Drop test/Jump down test, VEMP, OVAR response	Rotarod, Zebrafish Active Posturography (Zap); Floating Platform Tests–Postural sway–measured by Center of Pressure (COP) Assay (=COP), Righting reflex, VEMP, OVAR response, Active vs. Passive motion on vestibular nucleus neurons, Mid-air righting reflex	(1) Robotic simulations (2) What happens in a more regular schedule? (3) What are the effects of recurring cycles of sleep deprivation? How do they recover? How does it affect performance? We need biomarkers for that.	(1) Sleep loss and circadian changes affect the sensorimotor and cognitive function. (2) Caffeine + light − effective countermeasures. (3) Primary task is not affected during sleep loss but the secondary tasks are. This should be considered for effects on operational performance.
**DNA damage**	DNA repair, DNA metabolic process, cellular response to DNA damage stimulus	Brain/other tissues: Staining with Anti-8-oxo-dg, 53bp1	Blood: DNA lesions via HPLC, 8-oxo dg, micronuclei, double strand DNA breaks, chromosomal aberrations/translocations, one carbon metabolites	Cell cycle checkpoint activation, DNA Repair, apoptosis,	Radiotherapy	Cognitive tests	Oxidative stress and inflammation related cognitive tests	Monitor the level of DNA damage over time- need astronaut data	(1) Since brain and neurons are not proliferative, DNA damage is might not be relevant in CNS. However, peripheral DNA damage is useful to studying the general diversity and individual differences of responses to radiation (again a surrogate, assuming that the brain will respond the same as the rest of the body). (2) Use baseline DNA damage as a predictor for responses to irradiation/spaceflight (astronaut panel pre/post flight). (3) Sleep deprivation exacerbates DNA damage in rats and humans. We cannot train/adapt to sleep deprivation. Note suggested markers for radiation dosage-bio-dosimetry: FLT3LG, SAA1, C3, VCAM
**Blood brain barrier permeability**	Inflammation, one carbon metabolism	CSF: Albumin [150], Brain IHC—Aquaporin 4 [151], IHC, MMP-9, long-term microglial activation, astrocyte morphology, Endothelial cells, Somatic mosaicism	Blood: Occludin, c-Fibronectin, Ubiquitin carboxyl-terminal hydrolase isozyme L1, S100 calcium-binding protein B, Circulating brain microvascular endothelial cells ([150], stroke research), Corticosterone MMP-9, Cell free DNA Imaging: fMRI, PET scan, free water MRI; Epigenetic clock (accelerated aging).	Endothelial activation, Systemic inflammation, Kynurenine pathway, Tight junction damage, Oxidative stress, glial activation, MAPK pathway, PKC pathway, degradation of basal lamina and ECM.	Inflammation, stroke, Alzheimer’s	Stress: Visual Analog Scale Depression: Beck Depression Inventory	Locomotor activity, open field, hole-board, and grip strength tests, anxiety, and depressive behaviors	(1) Is BBB function altered in astronauts on ISS (or Artemis) missions? (2) Study the glymphatic system-removal of solutes from the brain across the BBB. (3) Need to understand the association of MMP9, occludins, S100, etc. with drainage of BBB. What is the physiological relevance? Glymphatic system is important for sleep as well. (4) Mutations, mosaicism etc. will affect the endothelial cells and may cause BBB leakiness, leading to physiological effects. (5) Association of sleep with debris clearance. Amyloid clearance from the brain occurs during sleep → relevance to both sleep/circadian and glymphatic system. (6) Astrocyte morphology—unexplored. Astrocyte expressing AQP4 would be important for glymphatic system. (7) Epigenetic clock measurements in astronauts and related to time in space etc. Or deep space to look at age acceleration (8) Development of rodent in vivo imaging technologies for BBB integrity. (9) Radiation induced senescence and functional readout in brain—glial cells, epithelial cells, somatic mosaicism	(1) Circadian changes in astronauts (avg. sleep 6 h. though allocated 8–9 h) can add more stress. (2) Epigenetic and aging association [152]. Easily conducted. (3) DNA methylation observed in radiation and inhibition on global level can mitigate hypermethylation related cognitive deficits.
**Vasculature**	Blood vessel development, heart development	Adhesion molecules (VE-cadherin), tight junction proteins (Claudin 3, 5, 12, Occludin), Zo-1, MMPs	Blood: Endothelial function markers (serum nitric oxide, tetra- and dihydrobiopterin (BH4) and (BH2), soluble intercellular adhesion molecule-1 (sICAM-1), soluble vascular cell adhesion molecule-1 (sVCAM-1), endothelin-1, asymmetric dimethylarginine (ADMA), L-arginine, formate, and soluble E-selectin. Imaging: fMRI, PET scan. Noninvasive peripheral arterial tonometry (PAT) technology can be used to assess the reactive hyperemia index (RHI) and the augmentation index [153]; Vascular damage MRI measures: Cerebrovascular reactivity (CVR) (Pre and post flight): Present with CO_2_ challenge; Free water. Plasma: Placental growth factor (PIGF), IL-8; VEGF-D; CSF: PIGF; IL-8	Adherens junction, Endothelial activation, systemic inflammation, oxidative stress, hypoxia	Inflammation, stroke, Alzheimer’s	Stress: Visual Analog Scale Depression: Beck Depression Inventory	Locomotor activity, open field, hole-board, and grip strength tests, and depressive behaviors	(1) What are the biochemical underpinnings of the thrombotic events seen inflight? (2) Also missing are chronic vascular injury markers. This biomarker has gained rapid adoption in many fields in the last few years. (3) Lack of cerebrovascular reactivity MRI data pre and post flight (4) Lack of 7T MRI for perivascular spaces (5) How do the biomarkers for vascular cognitive impairment change in astronauts? (6) Developing computational modeling of vascular changes?	Topological difference in vasculature and its susceptibility towards the various stressors
**miRNA regulation**	Transcriptional regulation		Serum: miR-383-5p [154]	Transcriptional regulation		Cognitive tests	Cognitive tests		
**Circadian Phase (sleep, sleepiness, performance impairment, immune function, endocrine function, bone metabolism, reproductive function)**			Lipidomics, metabolomics, transcriptomics, proteomics		Accident, injury (short-term/immediate); cardiometabolic and neurological disorders, compromised immunity (long-term)	Cognitive tests	Cognitive tests	Candidates identified; operational validation required	(1) Currently blood-borne but development of urinomics, saliva and breath matrices ongoing; (2) Can predict several days in advance; single vs. multiple samples. (3) Model organism—consideration of diurnal model over nocturnal. Marmoset? Indian palm squirrels?—restarting and reinventing the wheel? (4) Consistency in animal models and standardization in measurement. (5) Primary task is not affected during sleep loss but secondary tasks are. (Considered for operationally relevant performance)
**Neuronal and brain Damage Markers**	Blood: neurofilament, tau, abeta1-42, common pathology radiation and AD biomarkers (need to be explored)								Note suggested markers: NAA/Creatine ratio

## Data Availability

Not applicable.

## Abbreviations

5-HT	5-hydroxytryptamine
5MTHF	L-Methylfolate
8-oxo-dG	8-Oxo-2′-deoxyguanosine
Ach	Acetylcholine
AOP	Adverse Outcome Pathways
AQP-4	Aquaporin-4
ARC	Ames Research Center
BBB	Blood Brain Barrier
BDNF	Brain-derived Neurotrophic Factor
BMed	Behavioral Medicine
CBS	Central Nervous System, Behavioral Medicine, and Sensorimotor
CNS	Central Nervous System
COX-2	Cyclooxygenase-2
CSF	Cerebrospinal Fluid
DA	Dopamine
DMN	Default Mode Network
EEG	Electroencephalogram
Flt-1	Fms Related Receptor Tyrosine Kinase 1
fMRI	Functional Magnetic Resonance Imaging
GFAP	Glial Fibrillary Acidic Protein
GI	Gastrointestinal
HNE	4-hydroxynonenal
HRP	Human Research Program
ICAM-1	Intercellular Adhesion Molecule 1
IL-15	Interleukin-15
IL-4	Interleukin-4
ISS	International Space Station
JSC	Johnson Space Center
MAPK	Mitogen-activated Protein Kinase
MDA	Malondialdehyde
MMP-9	Matrix Metallopeptidase 9
MMPs	Matrix metalloproteinase
MRI	Magnetic Resonance Imaging
MTSBI	Model Translation & Space Biology Integration
NFKb	Nuclear Factor kappa B
NHP	Non-human Primates
NIRS	Near-Infrared Spectroscopy
PI	Principal Investigator
ROS	Reactive Oxygen Species
S100b	S100 Calcium Binding Protein B
SM	Sensorimotor
TIM	Technical Interchange Meeting
TNF	Tumor Necrosis Factor
TREM	Triggering Receptor Expressed on Myeloid cells
TRR	Transthyretin
UCSF	University of California San Francisco
USRA	Universities Space Research Association
USUHS	Uniformed Services University of the Health Sciences
VCAM-1	Vascular Cell Adhesion Molecule 1
VOR	Vestibular-ocular Reflex
YKL-40	Chitinase-3-like protein 1
ZO-1	Zonula occludens-1

## Appendix A. Agenda of Meeting

A NASA translational working group TIM titled Circuits and Biomarkers of the Central Nervous System Relating to Astronaut Performance (Biomarker TIM) was held virtually between 21–25 September 2020, and was supported by the NASA HRP’s Human Factors and Behavioral Performance Element in conjunction with Space Radiation Element and the Human Health Countermeasures Element. The goals of this Biomarker TIM were to (1) identify relevant brain regions, neural circuits, functions, and associated biomarkers that relate to operationally relevant performance and (2) identify any critical needs for new biomarker knowledge (“gaps”) that can be filled by additional focused and translational animal experiments that include a plausible pathway toward eventual biomarker validation in humans.

Deliverables addressing these goals may ultimately inform countermeasure strategies to maintain performance standards and identify performance limits for astronauts. To address the goals, 22 extramural experts from 19 academic institutions and 26 intramural experts from various NASA centers contributed to 15 talks reviewing findings from biomarker research on animals and humans in response to terrestrial and spaceflight stressors, and then participated in virtual thematic breakout sessions to systematically and qualitatively review biomarkers and associated brain circuits for 30 cognitive or behavioral constructs or physiological responses. The topics of the breakout sessions were sensorimotor influences (Group 1), behavioral medicine influences (Group 2), and integrated approaches to understanding operationally relevant performance (Group 3), and respective behavioral constructs listed in Table A1. Before the TIM, a portfolio of documents and scientific literature was shared with participants to frame the workshop and help the participants prepare.

**Table A1:** List of behavioral constructs for discussion groups.

Sensorimotor	Behavioral Medicine	Integrated Approaches: Physiological Responses
Visual	Memory	Neuroinflammation
Spatial Orientation	Attention and Dual Tasking	Neurotransmitters
Vestibular	Executive Function	One-Carbon Metabolism
Proprioception	Working Memory	Oxidative Stress
Hearing	Learning and Plasticity	Synaptic Plasticity and Neurotrophic Factors
Motion Sickness	Social Processes	Vestibular and Sensorimotor alterations
Smell and Taste	Individual Behavioral States	DNA Damage
Postural Control and Balance	Arousal and Regulatory	Blood Brain Barrier Permeability
Locomotion	Emotional Regulation	Vasculature
Fine Motor Control	Risk Taking/Tolerance	miRNA Regulation
Perception	Stress	Circadian Phase
Gaze		Neuronal Damage
Pain		

## Appendix B. Organizers & Participants

*Lead Organizers*

Joshua Alwood, PhD, NASA ARC

Ajitkumar Mulavara, PhD, KBR

*Organizer Team*

CBS/Johnson Space Center

Jayati Roy Choudhury, PhD, MEI

Kerry George, KBR

Jimmy Zaid, MEI

MTSBI/NASA Ames Research Center

Jared Broddrick, PhD

Egle Cekanaviciute, PhD

Janani Iyer, PhD, USRA

Laura Lewis

Siddhita D. Mhatre, PhD, KBR

April Ronca, PhD

Marianne Sowa, PhD

*Participants*

Group 1: Sensorimotor Influences on Operational Performance

**Leads**

Susanna Rosi, PhD, UCSF

Mark Shelhamer, ScD, Johns Hopkins University

**Facilitator**

Scott J. Wood, PhD, NASA JSC/Azusa Pacific University

**Expert Observers**

Millard Reschke, PhD, NASA JSC

Meghan Downs, PhD, NASA JSC

Sudhakar Rajulu, PhD, NASA JSC

Jeffrey Somers, PhD, NASA JSC

**Science Team**

Afshin Beheshti, PhD, KBR/Broad Institute

Kathleen Cullen, PhD, Johns Hopkins University

Sandeep Robert Datta, MD, PhD, Harvard University

Lisa Giocomo, PhD, Stanford University

James Lackner, PhD, Brandeis University

Gregory Nelson, PhD, Loma Linda University

Group 2: Behavioral Medicine Influences on Operational Performance (includes Cognition)

**Leads**

Catherine Davis-Takács, PhD, USUHS

David Dinges, PhD, University of Pennsylvania

**Facilitator**

Pete Roma, PhD, KBR

**Expert Observers**

Gillés Clement, PhD, KBR

Tim Macaulay, PhD, KBR

Sara Whiting, PhD, KBR

Erin Flynn-Evans, PhD, MPH, NASA ARC

Gary Strangman, PhD, Massachusetts General Hospital/Harvard Medical School

**Science Team**

Amelia Eisch, PhD, University of Pennsylvania

Thomas Jhou, PhD, The Medical University of South Carolina

Rachel Seidler, PhD, University of Florida

Steven Siegel, MD, PhD, University of Southern California

Andy Wyrobek, PhD, Lawrence Berkeley National Laboratory

Group 3: Integrated biomarkers and pathways relating to Operational Performance

**Leads**

Vivien Mao, MD, Loma Linda University

Rajeev I. Desai, PhD, Harvard Medical School/McLean Hospital

**Facilitators**

Ajitkumar Mulavara, PhD, KBR

Joshua Alwood, PhD, NASA ARC

**Expert Observers**

Honglu Wu, PhD, NASA JSC

Lisa Carnell, PhD, NASA Langley Research Center

Satish Mehta, PhD, KBR

Sara Zwart, PhD, KBR

**Science Team**

Janet Baulch, PhD, University of California, Irvine

Sylvain Costes, PhD, NASA ARC

Brian Crucian, PhD, NASA JSC

Daniel Geschwind, MD, PhD, University of California, Los Angeles

Steven Lockley, PhD, Harvard University

Scott M. Smith, PhD, NASA JSC

Patrick Stover, PhD, Texas A&M University

Donna Wilcock, PhD, University of Kentucky
